# MemPrO: A Predictive Tool for Membrane Protein Orientation

**DOI:** 10.1021/acs.jctc.5c01433

**Published:** 2025-12-23

**Authors:** Matyas Parrag, Phillip J. Stansfeld

**Affiliations:** † School of Life Sciences, 2707 University of Warwick, Coventry CV4 7AL, U.K.; ‡ Department of Chemistry, 2707 University of Warwick, Coventry CV4 7AL, U.K.

## Abstract

Membrane proteins play a vital role in numerous cellular processes, including ion transport, intercellular communication, and antibiotic resistance. Ensuring their accurate orientation within lipid bilayers is essential for reliable molecular simulations and structural analyses. We introduce MemPrO, a robust tool designed to orient a wide range of membrane-associated proteins with precision. While existing tools perform well in standard scenarios, they often struggle with complex configurations such as multibilayer systems or peripheral membrane proteins. MemPrO addresses these limitations by delivering detailed orientation data for proteins in both single and double membrane environments. In addition to mapping spatial arrangements relative to lipid bilayers, MemPrO incorporates advanced analytical features, including membrane curvature analysis. It also extends its capability to predict the position and orientation of the bacterial cell wall. By integrating these features, MemPrO significantly enhances our ability to model and interpret the intricate architecture of cellular envelopes. This supports both fundamental research and the development of novel therapeutic strategies through a deeper understanding of membrane–protein interactions.

## Graphical abstract

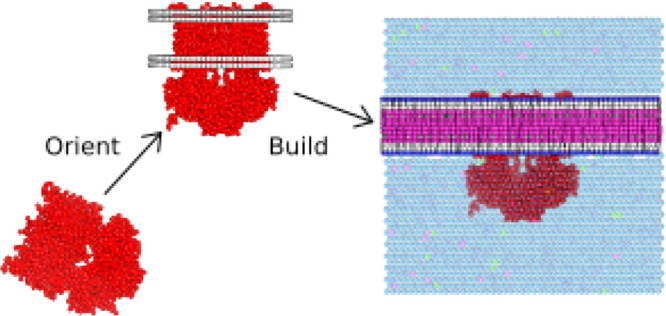

## Introduction

The cell envelope is a fundamental component of cellular life, comprising a diverse array of proteins essential for survival and function.^1^ These proteins perform critical tasks such as defending against external threats, maintaining structural integrity, and mediating processes such as cell division and nutrient transport.^2–4^

The study of membrane proteins is key to understanding cellular behavior and the mechanisms that underpin interactions with the environment, including responses to antimicrobial agents.^2–5^ Computational methods, particularly molecular dynamics (MD) simulations, are widely used to investigate membrane proteins embedded within lipid bilayers.^6,7^ These proteins adopt specific orientations within the membrane, driven by the distribution of hydrophobic residues across their surface. Determining this orientation via simulation, however, can be computationally demanding and complex, underscoring the need for efficient and accurate predictive methods.

Established tools such as OPM/PPM^8,9^ and MemEmbed^10^ have proven highly effective in orienting proteins within membranes and have become integral to many structural workflows, including MemProtMD.^6,11^ Their robustness and ease of use make them valuable resources for a wide range of membrane protein systems. However, certain complex scenarios, such as multibilayer arrangements, peripheral membrane proteins, or highly curved membrane geometries, can pose significant challenges. Additionally, while these tools provide reliable orientations, there remains a need for streamlined approaches that also generate lipid bilayers tailored for molecular dynamics (MD) simulations. Addressing these gaps presents an opportunity for further developments in this area.

The recent development of AlphaFold^12^ has revolutionized structural biology by enabling high-throughput and accurate prediction of protein structures. This breakthrough has, in turn, intensified the demand for cost-effective and reliable tools to support downstream stages of structural and functional analysis. In response to this need, we present MemPrO, a novel method designed to advance our understanding of protein–membrane interactions. MemPrO enables the high-throughput and automated construction of membrane protein systems, significantly accelerating large-scale simulation workflows. It also offers the flexibility to assemble complex membrane models by interfacing with *insane*,^13^ making it well-suited for establishing studies of specific protein–membrane interactions.

An automated setup of CG systems from an orientated protein is important for efficient simulation workflows.^6,11^ MemPrO incorporates Insane4MemPrO, a CG system builder based on *insane*,^13^ to automatically build CG systems with solvent, ions, lipids, and proteins. For single membrane systems, Insane4MemPrO uses methods from *insane*. However, for more complex systems, such as double membrane systems and curved membranes, several extensions to this methodology were implemented. Insane4MemPrO includes additional features that were not explicitly developed for MemPrO, namely the building of micelles around the hydrophobic regions of proteins and the construction of double membrane systems.

An effect of having two membranes is the creation of two disjoint water compartments separated by the membranes. This can create issues with the pressure equilibration between the compartments. When simulations of such systems are run, it is recommended to include a pore protein or an artificial pore in one of the membranes to allow the pressure to equalize between compartments. To address this further, additional modifications were implemented to bring the initial pressure closer to 1 bar and to allow for the assignment of ion concentrations and charges to each compartment individually.

The peptidoglycan cell wall is a large meshlike macromolecule found in many bacteria. In Gram-negative bacteria, it forms a relatively thin layer located in the periplasmic space between the inner and outer membranes. In contrast, Gram-positive bacteria possess a much thicker peptidoglycan layer situated outside the cytoplasmic membrane.^1^ This structure is composed of repeating murein subunits, each consisting of *N*-acetylglucosamine (NAG), *N*-acetylmuramic acid (NAM), and a short peptide chain attached to the NAM residue. These subunits polymerize into glycan strands, with NAM from one unit linking to NAG in the next. The peptide chains may remain free or form cross-links with peptides on adjacent strands, creating a highly cross-linked, mesh-like network that provides structural integrity to the bacterial cell wall.^14^

Proteins that traverse through the periplasm also pass through the PG layer. MemPrO provides predictions of where the PG layer may be positioned with respect to such a protein, and therefore, Insane4MemPrO has been extended further to enable the construction of a PG layer between the two membranes.

## Methods

### Initial System Setup

#### Lipid Bilayer and Solvent

The approach in MemPrO focuses on isolating only the essential elements of a coarse-grained (CG) protein description that are critical to determining the protein orientation. In CGMD simulations, interactions between CG beads are described by Lennard-Jones (LJ) radial potential curves, which are defined as

$$U_{\epsilon ,\sigma }\left(r\right)=4\epsilon \left(\frac{\sigma^{12}}{r^{12}}-\frac{\sigma^{6}}{r^{6}}\right)$$ 1

for parameters ϵ and σ.

For a central CG bead on the surface of the protein, the time-averaged density of environmental CG beads will peak at the distance *d*, corresponding to the minima of the LJ interaction. Since the lipids and solvent CG beads are highly mobile on the protein orientation time scale, we can treat the environmental CG beads as existing only at *d* for the purpose of orientation.

Hence, for a given CG bead, A, a cost can be assigned to a CG bead, B, which is in the local environment of A, where the cost is equal to the minimum value of the potential function of LJ between A and B. The minimal value of *U*_ϵ,σ_(*r*) is −ϵ. Define this minimal value as ϵ­(*T*_A_,*T*_B_) where *T*_A_ and *T*_B_ are the bead types of A and B*,* respectively. The parameters for the LJ functions between two CG beads are taken from Martini 3,^15^ although, in theory, any coarse-grained force field can be used within MemPrO.

Let B be a probe CG bead. B is either in the solvent, in the lipid bilayer, or on the boundary, depending on its position *Z*, where the *Z* axis is defined to be normal to the membrane. This will determine the type of bead, *T*_B_(*X*), of *B* at position *X*. We can use this information to construct a mean field potential, *M*_*T*_A__(*X*), as the cost ϵ­(*T*_A_,*T*_B_(*X*)) for each *T*_A_.

For a single CG bead, the contribution to the potential is calculated from an approximation of the local environment. The local environment of a bead, A, is constructed by placing beads, *B*_*i*_, around A such that they are evenly distributed on the surface of a sphere. The total score of A is then

$$\sum^{i}(M_{T_{A}}(X_{i}))$$ 2

where *X*_*i*_ is the position of *B*_*i*_.

As there is only a finite number of *B*_*i*_, moving A through the membrane would result in steps arising from the transition of each *B*_*i*_ between the solvent and lipid. The resulting function would therefore have zero-gradient regions, making gradient-based minimization an impossibility. To rectify this, a sigmoid function

$$s(x)=\frac{1}{1+e^{-x}}$$ 3

is used to smooth *M*_*T*_A__, removing the zero-gradient regions. The result of this process, *M*_*T*_A__′, is shown in Figure 1b. *M*_*T*_A__′ will still have some regions with near-zero gradient far from the boundary region, but since there will always be a CG bead near the boundary, unless the protein is fully in solvent, there will always be a large gradient to drive minimization. The local environment also includes the protein itself. If a bead *B*_*i*_ is located within the protein, its contribution to the mean field potential is ignored.

**Figure 1:**
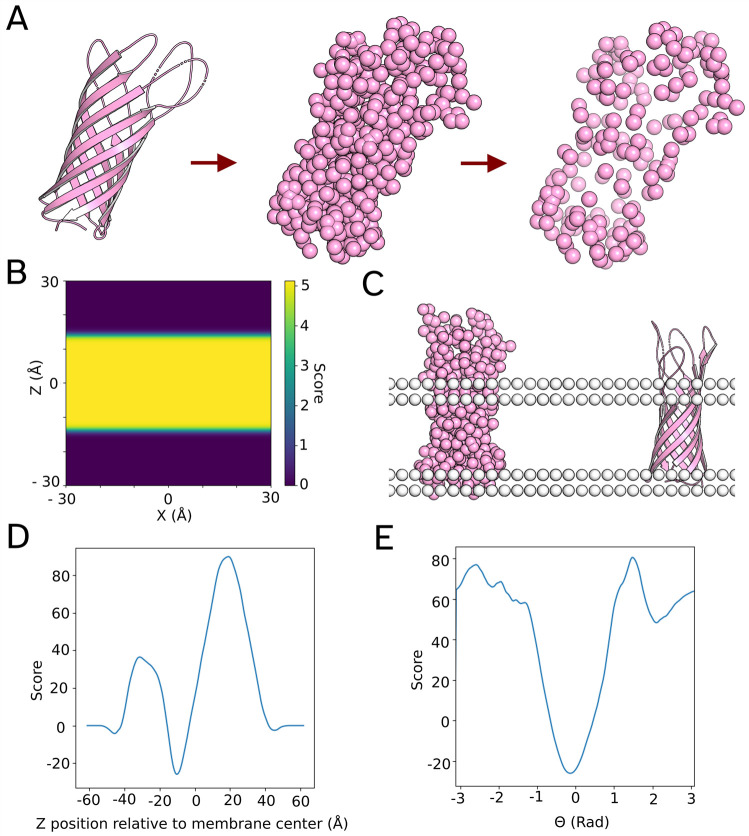
A summary of the MemPrO method for single membrane proteins. (A) The preprocessing of the input protein, for CG inputs, starts at step 2. (B) The mean field at a cross section of the membrane for a CG bead contained in Arginine and Lysine. Purple indicates a low energy, while yellow indicates a higher energy. This particular CG bead prefers to be in solvent and not in the core of the membrane. (C) The output of MemPrO, the format of the output, is the same as the input. (D) The potential energy curve *y* = *P*(*z*,θ,ϕ) where (θ,ϕ) is fixed for the protein 1BXW. There is a large minima that roughly corresponds to a *z* such that the hydrophobic region of the protein is within the membrane core. (E) The potential energy curve *y* = *P*(*z*,θ,ϕ) where (*z*,ϕ) is fixed.

#### Setting Up the Protein

Since MemPrO relies on coarse-grained (CG) representations to compute the orientation potential, proteins must first be converted to CG models. This is accomplished using Martinize2.^16^ During the orientation step, the protein is treated as a rigid body, meaning that the geometry optimization features of Martinize2 are not required. As a result, atomistic structures can be rapidly coarse-grained using a streamlined preprocessing step, allowing users to proceed directly to orientation without the need for extensive structural refinement.

To improve the efficiency of calculating the total protein score, all core beads, representing residues that do not interact with the environment, are removed. This is accomplished using a surface-finding algorithm specifically developed for MemPrO. Typically, surface beads are defined as those with a solvent-exposed area exceeding a specified cutoff value. However, for the MemPrO orientation, the surface bead set does not need to be exact. This allows for the use of a less accurate, but more computationally efficient algorithm.

The method used in MemPrO is as follows: A bead B is placed sufficiently far from the protein and moved along a line toward a bead A contained in the protein. The first protein bead with which B comes into contact is considered a surface bead. This process is repeated in batches until the number of new surface beads is below a threshold. The effects of the randomness inherent in the method are investigated in the Supporting Information (SI) Section 2.

### Evaluating the Potential

The total score is the sum of the contributions of each CG bead, where the score of each CG bead is a function of its position. Consequently, the total score can be expressed as a function *P*(*X*,Θ), where *X* represents the position, and Θ represents the rotation of the protein. Since translation parallel to the membrane does not affect the potential, the only positional coordinate needed is *z*, which is defined as the insertion depth of the protein into the membrane core. The direction of the protein can be described by two angles, θ and ϕ. However, this representation can introduce artificial barriers during minimization, as two configurations that are spatially close may not be near each other in terms of θ and ϕ. To resolve this, the direction is instead represented as a 3-vector *v*, with the protein’s rotation about *v* described by a 2-vector *w*. Let *P*(*z*,Θ) denote the total score of the protein, where *z* represents the insertion depth, and Θ is the 5-vector that describes the rotation. Here, Θ and the pair (θ,ϕ) will be used interchangeably to represent the rotation of the protein.

For charged CG beads, the contribution from the Coulomb interaction must be considered. This is calculated by approximating the membrane as charged sheets, corresponding to the planes described by the lipid head groups. The potential between a charged CG bead and each sheet is calculated by performing a numerical integration over a circle with radius equal to the cutoff distance. Investigation of orientations across a data set of proteins reveals that, on average, MemPrO performs best when assigning a charge of 0 to each sheet. This may be due to the sparsity and mobility of charged lipids within the membrane, resulting in a highly spatiotemporally varying charge. However, in cases where charged lipids are localized around the protein, this variance is greatly reduced, making a nonzero charge more appropriate. This is typically observed with peripheral membrane proteins that associate with the membrane through electrostatic interactions.^17^

### Minimization Scheme

#### Grid

The function *P*(*z*,Θ) generally has several local minima, and it is not guaranteed that the global minimum corresponds to the desired orientation, nor that there is only one valid orientation. To correctly identify the natural orientation(s), all local minima must be found and reported. For each local minima, *L*_*i*_, there is a set *S*_*i*_ of starting orientations that converge to *L*_*i*_. By running several minimizations in parallel, each starting from a different initial orientation, all *L*_*i*_ with sufficiently large *S*_*i*_ can be identified. MemPrO will report every local minimum found, with the initial orientation grid, *G*, distributed uniformly to ensure no bias toward a particular *L*_*i*_.

As mentioned, *G* is a set of initial configurations for which minimization will be performed. The construction of *G* is crucial: it must sample as many *S*_*i*_ as possible while remaining computationally tractable and unbiased. Let (θ_*i*_,ϕ_*i*_,*z*_*i*_) ∈ *G*, (θ_*i*_,ϕ_*i*_) represents the direction of the protein, which can be translated into the 5-vector description before minimization. (θ_*i*_,ϕ_*i*_) is chosen according to a Fibonacci spiral lattice, with θ in the range $\left[0,\frac{\pi }{2}\right]$ and ϕ in the range [0,2π]. To maintain a manageable size of *G* due to the higher dimensionality, *z*_*i*_ is not varied independently but rather depends on (θ_*i*_,ϕ_*i*_).

#### Initial Insertion Depth

As *z*_*i*_ depends on (θ_*i*_,ϕ_*i*_), it can be denoted as a function *Z*(θ_*i*_,ϕ_*i*_). An integral membrane protein will typically contain a band of hydrophobic residues, which resides within the hydrophobic core of the bilayer.^6,11^ Therefore, *Z*(θ_*i*_,ϕ_*i*_) is chosen to be an approximation of the *z* position of the hydrophobic band for a given rotation (θ,ϕ) of the protein.

*Z*(θ_*i*_,ϕ_*i*_) is calculated by first assigning to each bead, b, a value *h*_b_ corresponding to the interaction strength between b and water. A second value, *h̅*_b_, is then assigned to each bead b, where *h̅*_b_ is the average of the set {*h*_b_|*b* ∈ *B*}, with *B* being the set of all beads within 20 A of b. Finally, the positions *X*_b_(θ,ϕ) of those *b* with *h̅*_b_ > *C*, where *C* is a cutoff value, are averaged to give *Z*(θ_*i*_,ϕ_*i*_).

Not all membrane proteins contain such a hydrophobic band,^17^ so an alternative approximation *Z*′(θ_*i*_,ϕ_*i*_) is implemented. *Z*′(θ_*i*_,ϕ_*i*_) is chosen such that the potential *P*(*Z*′(θ_*i*_,ϕ_*i*_),θ_*i*_,ϕ_*i*_) is minimal and is found through a grid search on *z*. For integral membrane proteins, the approximation *Z* ^*′*^(θ_*i*_,ϕ_*i*_) may not always be close to *Z*(θ_*i*_,ϕ_*i*_), since while *Z*(θ_*i*_,ϕ_*i*_) will always be near a local minimum, it is not necessarily the global minimum. Therefore, *Z*(θ_*i*_,ϕ_*i*_) is the default approximation, while *Z* ^*′*^(θ_*i*_,ϕ_*i*_) is only used in special cases, such as for peripheral membrane proteins.

#### Minimization

The minimization algorithm used in MemPrO is an implementation of AdaDelta^18^ in JAX.^19^ JAX is also used for the purpose of differentiating *P*(*z*,Θ) with respect to each independent variable. The code is heavily optimized for computational efficiency using JIT compilation provided by JAX.

After minimization, an optimal (*z*, θ, ϕ) is obtained for each configuration in *G*, the set of initial configurations. Let (*z*′, θ′, and ϕ′) represent the configuration, where the protein is reflected about the center of the membrane. Given a symmetric membrane, the following relationship holds:

$$P\left(z,\theta ,\phi \right)=P\left(z^{\prime },\theta^{\prime },\phi^{\prime }\right)$$ 4

To account for the positive inside rule, the inner leaflet of the membrane is assigned a slight negative charge. This modification alters the equality above to

$$P\left(z,\theta ,\phi \right)<P\left(z^{\prime },\theta^{\prime },\phi^{\prime }\right)$$ 5

if and only if the configuration (*z*, θ, ϕ) obeys the positive inside rule.^20^

### Postprocessing

Let *G*′ be the set of configurations obtained by minimizing all of the elements of *G*. *G*′ will contain a set of clusters, {*C*_*i*_}, each centered on a local minimum found. The clusters {*C*_*i*_} are determined through an iterative procedure. For each element *g*_*i*_ ∈ *G*′, if there exists an element *c* ∈ *C*_*j*_ for some *j*, where *g*_*i*_ is within a tolerance *t* of *c*, then *g*_*i*_ is added to *C*_*j*_. Otherwise, {*g*_*i*_} forms a new cluster. In practice, *t* can be small, as each cluster has a very low variance after sufficient minimization iterations.

The resulting set of clusters, {*C*_*i*_}, corresponds to a set of local minima {*L*_*i*_}, which can be determined by averaging all configurations in each *C*_*i*_. The set {*L*_*i*_} will include orientations that may not be biologically relevant, due to either a high potential or the shape of the minima. To filter out these orientations, {*L*_*i*_} is sorted with respect to *R*_*i*_, where *R*_*i*_ depends on the minimal value and depth of *L*_*i*_.

### Predicting Double Membrane Systems

The protocol can be modified to orient proteins that pass through two membranes, such as gap junctions or periplasm-spanning proteins. To achieve this, the mean field, *M*_*T*_A__′, representing the local environment, is adjusted to include two lipid regions for each *T*_*A*_. *M*_*T*_A__′ will now depend additionally on the distance, *D*, between the two membranes, hence the total score becomes *P*(*z*,Θ,*D*). As a result, each initial configuration (*z*_*i*_, θ_*i*_, and ϕ_*i*_) in *G* must also include *D*_*i*_. Varying (θ,ϕ) alongside *D* would prove too expensive, so *D* needs to be approximated, similar to the case for *z*. Details on how *D*_*i*_ is approximated can be found in the Supporting Information.

#### Peptidoglycan Layer Prediction

In Gram-negative bacteria, both an inner and an outer membrane are present, separated by a periplasmic space. Within this space lies the peptidoglycan (PG) cell wall.^21^ Determining the orientation of a protein that spans the periplasm can enable the prediction of the location of the PG cell wall.

To predict the placement of the PG cell wall, scoring function *P*_PG_(*z*), where *z* represents the position of the PG layer, must be constructed.

Let *N*(*T*_A_,*X*,*z*) be a mean field representing the PG layer, where for a bead A, *T*_A_ is the bead type, *X* is the position, and *z* is the placement of the PG layer. This field is constructed like *M*_*T*_A__′(*X*) in the standard method. *N*(*T*_A_,*X*,*z*) is built using Martini2 ^22^ parameters, as Martini 3 ^15^ parameters for PG were unavailable at the time of writing. The contribution to the total score for a given *z* is the sum of *N*(*T*_A_, *X*, and *z*) over all beads A. Let *L*(*z*) denote this sum.

For a lipid bilayer, it was found that Coulomb interactions do not contribute significantly to the final orientation. However, in the PG layer, the charged beads are immobile, making charge interactions critical. The PG layer and both membranes are modeled as charged sheets. The potentials between the PG layer, charged CG beads within the protein, and both membranes are calculated using numerical integration. Let *C*(*z*) represent the potential due to a charge with the PG layer at position *z*. The total score *P*_PG_(*z*) is then given by

$$P_{\mathrm{PG}}(z)=C(z)+L(z)$$ 6

The global minimum of *P*_PG_(*z*), denoted as *z*_PG_, represents the predicted position of the PG layer. *z*_PG_ is determined via a grid search on the variable *z*. Since external factors may influence the true position, the graph of *P*_PG_(*z*) is also provided to illustrate the scoring function along *z*.

#### Cross-Sectional Area in the PG Layer

MemPrO also outputs the cross-sectional area of the protein in the PG layer. As mentioned in the section ”Peptidoglycan layer prediction,” due to external factors, the correct placement of the PG layer is not necessarily the lowest minima, and so a graph of the cross-sectional area is outputted to capture all possible placements.

The cross-sectional area at a given distance along the protein is calculated as the area of the convex hull of the section of the protein passing through the PG layer. The convex hull is a good approximation of the area, as the structure of the PG layer will prevent it from filling out concavities.

### Global Curvature Prediction

Membranes are rarely truly planar, either in simulations or in nature.^6,11^ While small membrane deformations caused by proteins may not need to be present at the start of a simulation, larger deformations can create significant challenges when simulating planar membranes as the initial condition. For proteins that prefer highly curved membranes, determining the orientation can be significantly complex without accounting for curvature. OPM-PPM3 ^9^ performs global curvature prediction, which can aid orientation in such cases. We have therefore implemented a global curvature prediction in MemPrO.

As predictions are calculated with the protein in isolation, distinguishing between short-range local deformations and long-range global deformations is not always straightforward. Consequently, orientations with curvature should be treated as suggestions rather than as definitive predictions.

Global curvature is defined as a constant curvature, *c*, across the membrane surrounding the protein. *c* becomes an additional independent variable influencing the total score. The mean field is adjusted, as shown in Figure 4a, to account for varying *c*, and predictions are obtained by minimizing *c* alongside Θ and *z*. In all starting configurations, *c* is initially set to 0, as this is usually close enough to the true curvature for effective minimization.

### Building Simulation-Ready Systems

The method for constructing all systems, except micelles, is as follows. Each lipid is placed uniformly on a surface determined by user input. The position of each lipid is determined by the surface parametrization, and each lipid is oriented such that its normal vector aligns with the surface at that position.

#### Building Micelles

For micelle construction, a more complex algorithm is required, as the surface parametrization depends on the protein. The lipids are placed on a toroidal shell using the general methodology, but to form a micelle, this toroidal shell must follow the curvature of the protein. Additionally, the curvature may vary along the *z*-axis, and the curvature of the micelle must reflect this variation.

Initially, the protein is sliced to retain only the transmembrane region, which is obtained after the orientation. A Gaussian function, *G*_a_(*x*,*y*), is then placed at the *x*, *y* coordinates of each atom a. Let *S*(*x*,*y*) represent the sum of *G*_a_(*x*,*y*) values over all atoms *a*. The contours of *S*(*x*,*y*) follow the shape of the protein as desired.

A toroidal shell of lipids is composed of rings with varying radii and heights. Each ring is constructed by placing lipids along the contours of *S*(*x*,*y*) with the values of *S*(*x*,*y*) chosen to ensure that the radii, *R*, and heights, *z*(*R*), of all rings form a toroidal shell. Care must be taken to avoid clashes between lipids, as the curvature can be quite extreme.

#### Building the PG Layer

Initially, glycan strands are created with a length equal to the size of the cell. When a protein is present, NAG-NAM units that collide with the protein are removed. The distribution of glycan strand lengths varies greatly between different species of bacteria and even temporally within cells. The lengths of the glycan strands have a large impact on the mechanical properties of the cell wall,^23^ motivating the need to be able to control this parameter when building systems. The glycan strand length distribution can be controlled via a user-defined sum of Gaussians. To match this distribution, a Markov chain Monte Carlo (MCMC) simulation is used with moves corresponding to joining or breaking strands. The acceptance criteria are based on the Kullback–Leibler (KL) divergence of the new distribution from the target distribution and are as follows:

$$1-e^{K-K_{p}/Kt}$$ 7

where *K* is the KL divergence between the current and the target, *K*_p_ is the previously accepted KL divergence, and *Kt* is a constant. In small cells, matching the target exactly is very difficult, but there will generally be good agreement between the actual distribution and the target. After the MCMC, any strands that are still the length of the entire cell are broken in two. As there are periodic boundary conditions (PBC), this corresponds to a change from an infinite length to a finite one.

The second step is to generate cross-links between the NAM units. Each unit, *U*, is randomly assigned either a left or right direction, *D*_*U*_. Cross-links are formed by repeating the following: A random unit, *U*_1_, is selected, and a second random unit *U*_2_, is selected on an adjacent strand in the direction *D*_*U*_1__. A cross-link is formed with probability *P*_cl_, where *P*_cl_ is defined by the user. If a cross-link is not formed, the pair *U*_1_ and *U*_2_ will still be flagged as linked, so the pair is not selected for cross-linking again. The direction *D*_*U*_1__ is overridden with probability *P*_l_, where *P*_l_ is defined by the user as either up or down. In this case, a cross-link is formed between PG layers.

A single cross-link will form a dimer; however, it is theoretically possible for a third unit to link to this dimer, which will result in a trimer. This can occur many times, leading to higher order oligomers. The cross-linking procedure described above can also generate such oligomers with probability *P*_o_, where *P*_o_ is defined by the user. When forming cross-links, great care must be taken to avoid links that would be extremely energetically unfavorable or lead to steric clashes.

The result of the above procedure is a set of PG layers, *P*. Each layer will be slightly stretched due to the nature of the construction process. This can lead to unwanted compressive forces on other components of a simulation cell. To deal with this issue, simplified versions of each layer in *P* are created. Let *P*_s_ denote these simplified layers. A quick simulation using springs is run on each layer in *P*_s_ simultaneously. This allows the layers to relax to a more natural conformation. During the simulation, the total extensional and compressional forces on each bond are used to adjust the size of the simulation cell. The result is a fully relaxed set of simplified PG layers from which the PG layers can be rebuilt. When a protein is present, units are repelled from the protein, ensuring that there are no clashes between the protein and the PG layer.

A custom parameter (.itp) file for the PG layer is created for running simulations.

#### Obtaining Angle Parameters

Parameters were taken from ref 24 and adjusted for Martini 3. In previous work,^24^ no angle term was reported for the interpeptide link. This was calculated by tracking positions of CG beads superimposed over an atomistic simulation of a small section of a PG layer, following the procedure used in Vaiwala et al.^24^ CG parameters were adjusted to best match the resulting distribution. There are two angles associated with the interpeptide link. The angles and force constants calculated were 114°, 75 kJ mol^–1^ and 95°, 75 kJ mol^–1^.

## Results and Discussion

MemPrO was run with 36 starting configurations for 150 minimization iterations across a large number of proteins in various systems. The rank one output of MemPrO was compared to MemProtMD data as well as to other membrane orientation software to verify its accuracy. Further analysis of the method is provided in SI Section 2.

### Single Membrane Proteins

MemPrO was evaluated alongside OPM-PPM3 ^9^ and Memembed on a subset of the mpstruc^25^ database of membrane proteins. Since Memembed requires a separate flag for beta-barrel proteins, the data were divided into α-helical and beta-barrel proteins to more accurately evaluate performance. The local version of OPM-PPM3 was used with the membrane specified as an undefined membrane. The database consisted of 1175 α-helical and 194 beta-barrel transmembrane proteins.

Simulation snapshots from MemProtMD^6^ were used as the ”ground-truth” orientation. As simulations can be noisy, there is an inherent error associated with this ground truth; therefore, small deviations are not considered significant. MemPrO generates a set of initial configurations that uniformly samples the input space, always including the input orientation. To avoid potential bias, all input configurations were randomized. The impact of the initial configuration on MemPrO is further investigated in SI Section 2.

To evaluate accuracy, both the insertion depth and tilt angle were used. For each orientation, the error with respect to MemProtMD was calculated. The insertion depth of the protein depends on both the position of the protein and the lipid bilayer. Both positions are subject to noise in the simulation snapshot, introducing additional errors in the estimation of insertion depth deviation. The local version of the OPM-PPM3 automatically applies curvature to the membrane, which complicates direct comparison of insertion depth; thus, it is excluded from the analysis.

Figure 2 shows KDE graphs of angle and insertion depth deviations. For α-helical proteins, all methods perform very well, with no significant difference between them. Overall, MemPrO is shown to perform as well as the current state-of-the-art for the orientation of proteins within a single membrane.

**Figure 2:**
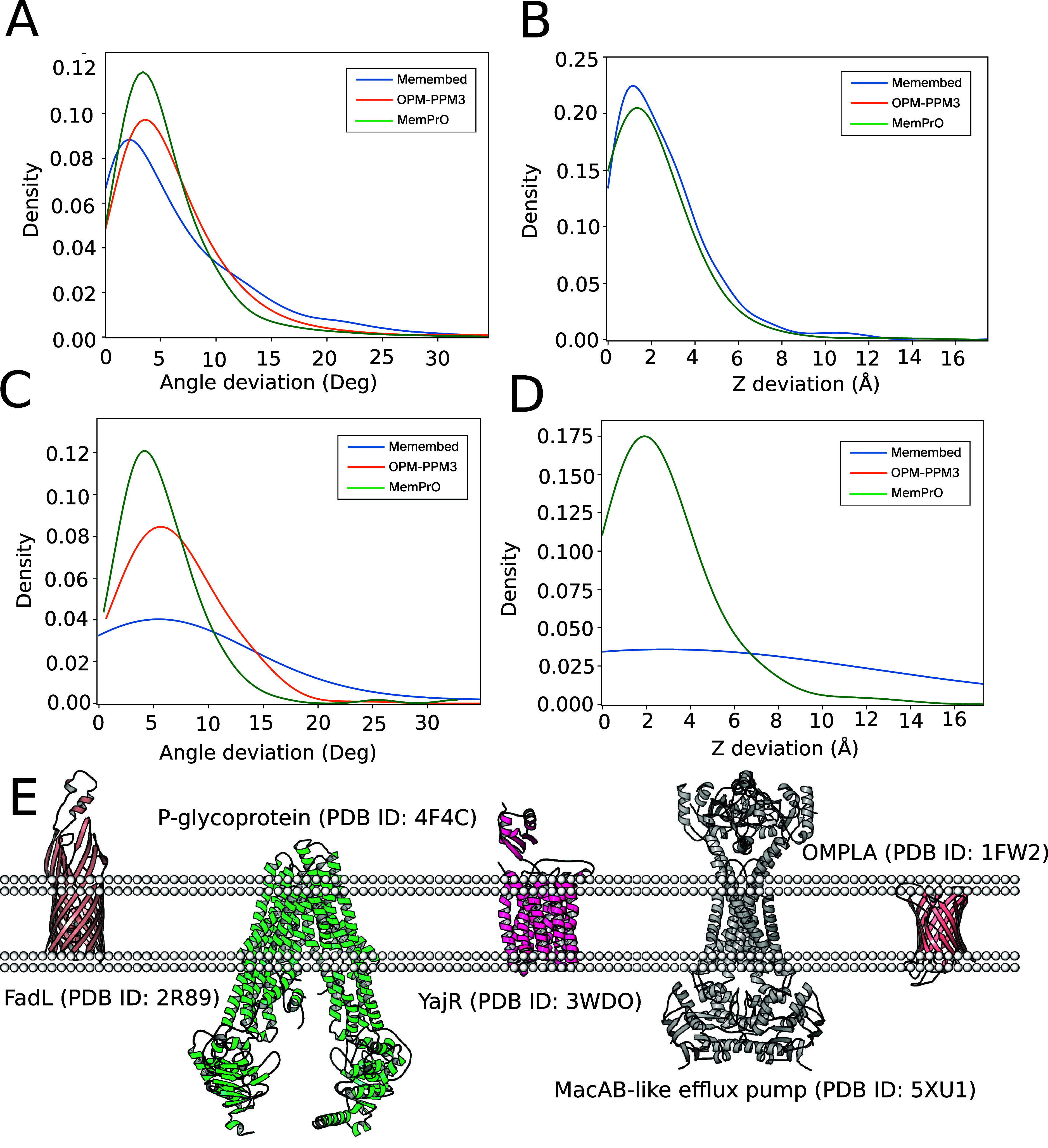
Comparison between methods on example proteins within a single membrane. (A) A KDE graph of angle deviations from MemProtMD for α-helical proteins. All methods perform well, with MemPrO showing the best average. (B) A KDE graph showing insertion depth deviation for α-helical proteins. Again all methods perform well. (C) As for (A) but for beta-barrel proteins. This time MemEmbed shows significant angle deviations, while the other two methods are similar to α-helical proteins. (D) As for (B) but for beta-barrel proteins. MemEmbed shows significant deviation. (E) Some examples of membrane protein orientation within a single membrane by MemPrO.

### Computational Efficiency

To assess the computational efficiency of MemPrO, the time taken to orient 500 proteins of varying sizes was recorded and compared to the local version of OPM-PPM 3.0 and Memembed for the same set of proteins. Each code was run assuming a standard desktop environment, which is to say, assuming access to approximately 9 CPUs. Figure 3A,C shows the average time to orient against the number of atoms in the protein to be oriented. The most lightweight of the 3 methods, Memembed, is also the fastest code by an order of magnitude. MemPrO is again an order of magnitude faster than OPM-PPM 3.0. Even when restricting MemPrO to a single CPU, it remains significantly faster than OPM-PPM 3.0.

**Figure 3:**
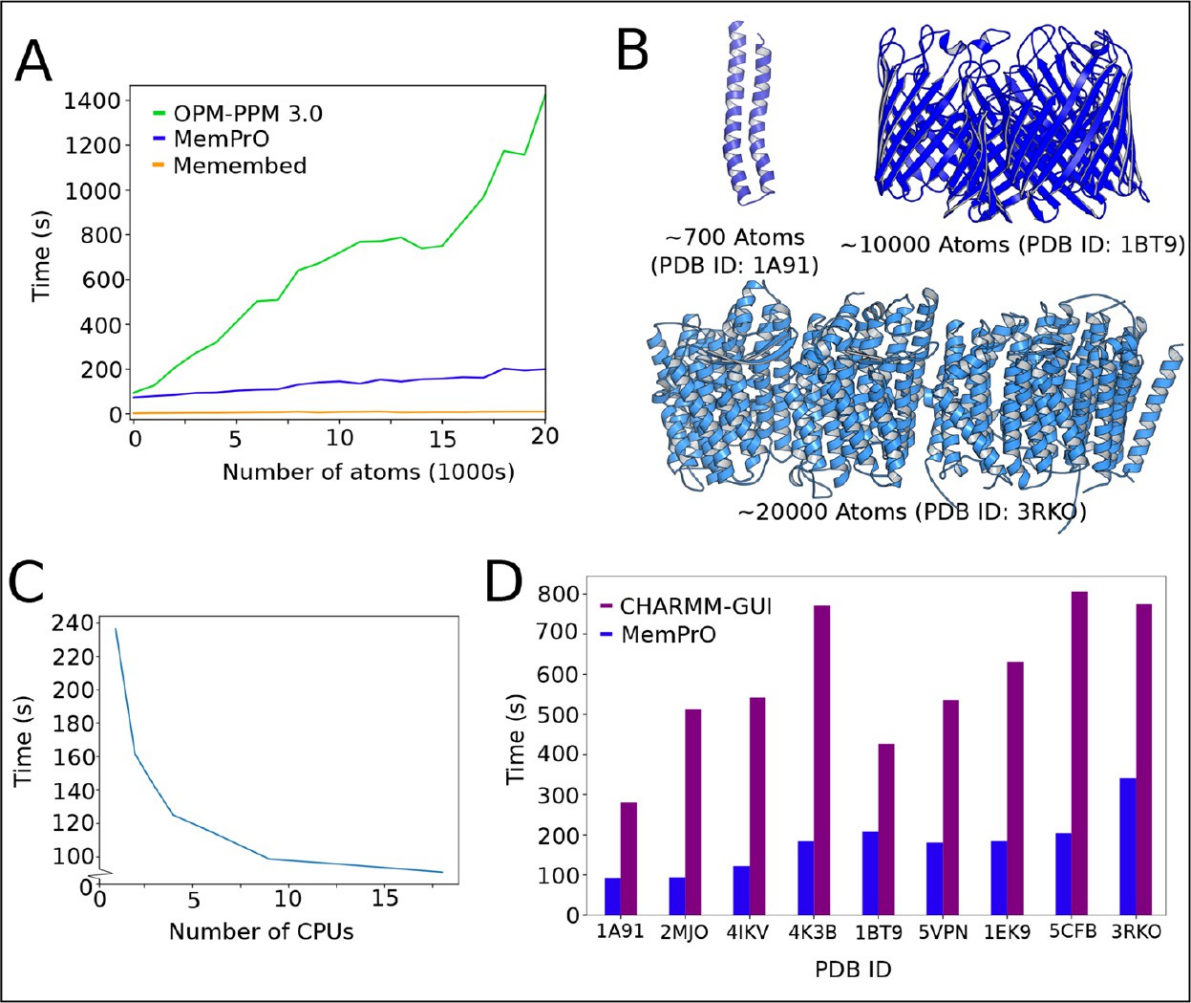
Computatinal efficiency of MemPrO, OPM, and Memembed. (A) Runtime against number of atoms for OPM-PPM 3.0, MemPrO, and Memembed. (B) Examples of proteins with ∼1000, ∼10,000, and ∼20,000 atoms. (C) Runtime against number of CPUs for MemPrO. This was preformed on the protein with PDB ID 3TT1, which has approximately 10,000 atoms. (D) Comparison of runtime for the full MemPrO workflow against CHARMM-GUI. Here CHARMM-GUI does not preform orientation, and as inputs must be atomistic, the run time of martinize2 is added to MemPrO for fair comparison. The high variance in CHARMM-GUI runtime is due to the difference in server load at the time of running.

Figure 3D shows how MemPrO scales with increasing CPUs. As much of the run time is due to JAX compilations, which are strictly serial, only approximately 67% of the code is parallelizable, which is reflected in the time to run in the high CPU limit. Despite increased runtime due to JIT compilations, the increase in speed greatly improves the efficiency of MemPrO.

Finally, the entire MemPrO workflow was compared to the CHARMM-GUI martini maker.^26^ CHARMM-GUI only takes an atomistic input and preforms coarse graining as part of the workflow, and as MemPrO is only intended to fit into a workflow and hence does not preform coarse graining itself, the runtime of Martinize2 was added to the runtime of MemPrO for the purpose of this comparison. Additionally, the CHARMM-GUI martini maker uses only a simple orientation method, which will not return a correct orientation in all cases. Other similar tools available from CHARMM-GUI use an OPM-PPM 2.0 to orient, but this was not available in this case. Figure 3E shows the runtime over several proteins of varying sizes. In each case, a simple POPE lipid bilayer is built around the protein, with system sizes matching as closely as possible between the two methods. MemPrO runs significantly faster than the CHARMM-GUI martini maker for all example proteins.

### Global Curvature Prediction

Determining global curvature from experimental data or simulation is very challenging, leaving few results with which to compare predictions. Despite this, it is possible to infer from simulations, or even attempts to orient without curvature, whether a protein would prefer a highly curved environment. Examples of such proteins are shown in Figure 4c,d; in both of these cases, orientation without the use of curvature results in orientations that would likely lead to an incorrect simulation.

**Figure 4:**
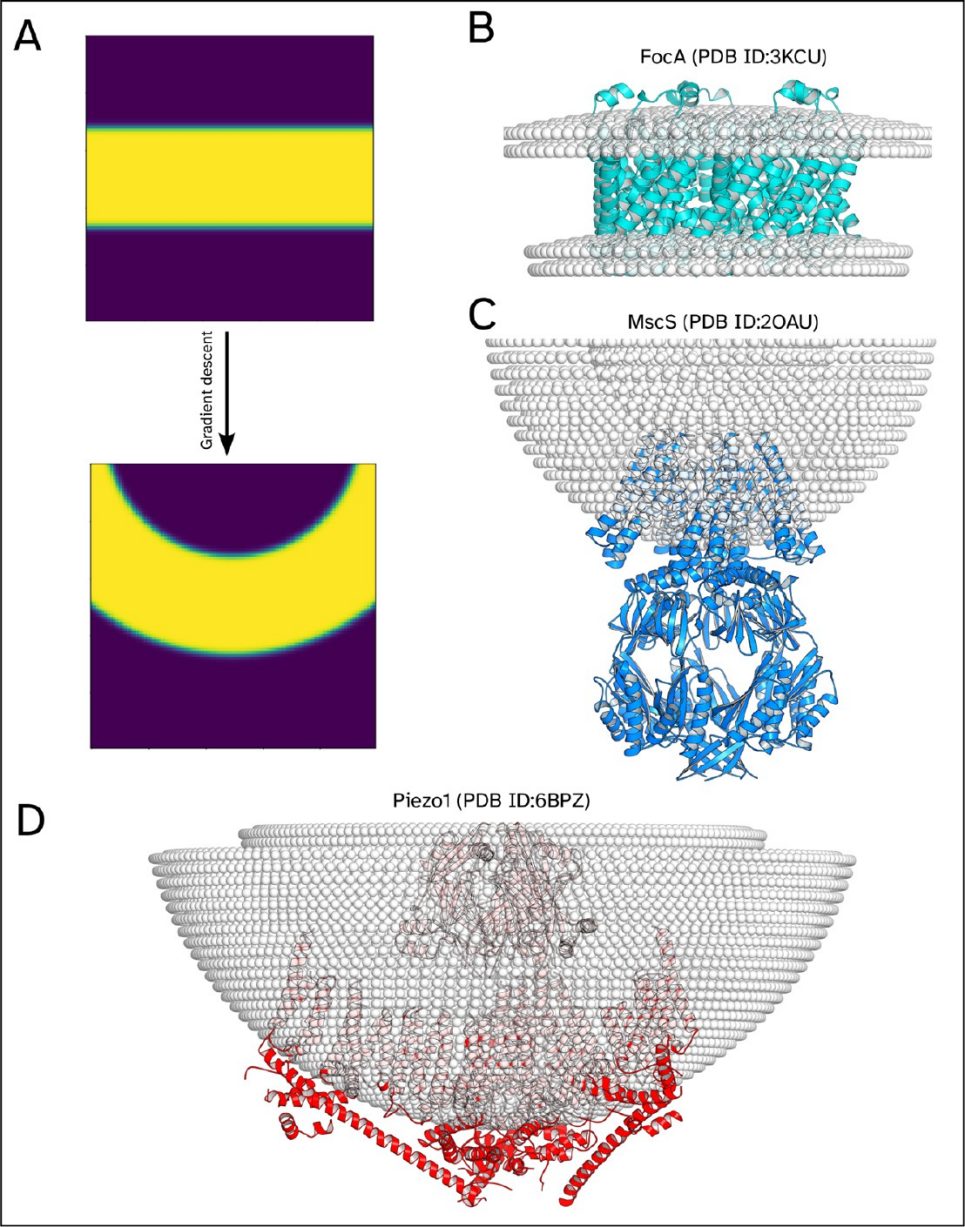
Some examples of global curvature prediction with an illustration of the modified method. (A) An cross-section of the potential field during minimization with global curvature. (B–D) Some examples are global curvature predictions. For 2OAU (C), the curvature may instead be local deformations as opposed to a more global curving as shown.

To better understand how global curvature impacts orientation, an analogy to fitting lines of best fit can be considered. Given a collection of points that sample a straight line with noise, a line of best fit can be calculated that will recover the original line with good accuracy. If instead the points sample a semicircle, the line of best fit will not be able to reflect the underlying pattern. If, however, the line of best fit is allowed to curve, then once again the original semicircle can be recovered. This corresponds to orientation by relating the collection of points to the hydrophobic core of the protein and the line of best fit to the position of the bilayer. When the global curvature predicted is small, as in Figure 4b, it can be difficult to determine if this is a true global curvature or the result of local deformation around the protein. Using the analogy, this is equivalent to fitting a curved line of best fit to noisy data, where it can be difficult to tell if curvature is due to noise or an underlying structure.

With this in mind, it is generally better to restrict the bilayer to be planar, thus avoiding errors caused by unnecessary curvature; however, allowing the bilayer to curve is essential to orienting some proteins. Whether or not curvature is being predicted, MemPrO will calculate the potential over a range of curvatures for the final orientation; this will highlight if a high curvature is preferred, allowing possible further orientation using curvature as required.

### Double Membrane Proteins

The orientations of some double membrane-spanning proteins are shown in Figure 5, and the placement of the PG cell wall for periplasm-spanning proteins is shown in Figure 5d. Currently, there are limited examples of double membrane proteins in the PDB, and there is little to no experimental or simulation data to verify their orientations; hence, no large-scale comparison is performed. Some verification is possible by looking at simulation data for proteins that are part of the larger overall complex, for example, in the case of the efflux pumps, the orientation of TolC can be used to verify the accuracy of the prediction at least partially.

**Figure 5:**
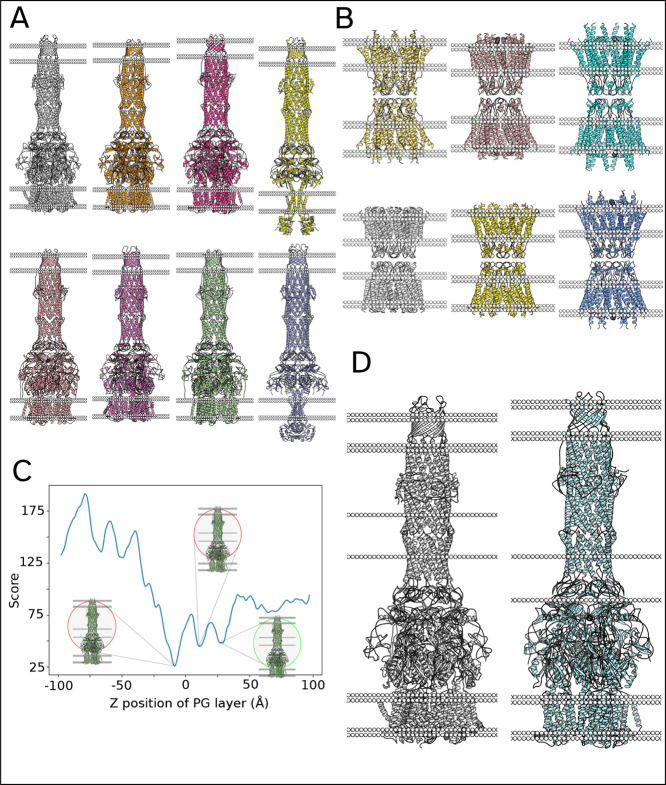
Some examples of double membrane (DM) orientation and placement of the PG cell wall. (A) Some examples of double membrane orientation for periplasm-spanning proteins (5O66, 6IOK, 6IOL, 5NIK, 6TA5, 65NG5, 6TA6, and 5NIL). (B) Some examples of DM orientation for gap junctions (2ZW3, 7ZIT, 7XQB, 6MHQ, 7XKK, and 7F93). (C) A graph of pseudo potential against placement of PG cell wall, which is an output of MemPrO. Global minimum does not correspond to what is expected for this protein, however one of the local minima does. (D) Examples of PG cell wall placement by MemPrO (5O66 and 6IOL).

The orientation of some double-membrane-spanning proteins can be inferred by orienting the upper and lower segments separately. Though this method would work in the examples shown in Figure 5a,b, it fundamentally assumes the orientation of the upper segment is independent of the orientation of the lower segment. The approach used in MemPrO is a more general and robust method that performs well in more eccentric cases. Further investigation of this can be found in the Supporting Information.

Prediction of the PG cell wall position relies solely on the protein in isolation. However, in nature, various external factors can influence the positioning,^21^ potentially causing a discrepancy between the predicted and actual positions. Typically, the true position corresponds to one of the local minima, as illustrated in Figure 5c. One of the major external factors is Braun’s lipoprotein (LPP), which is bound covalently to the PG layer and inserted into the outer membrane in certain species of bacteria. This acts as an anchor for the PG layer, effectively determining the position entirely.^21^ A good use case of this prediction method is when, though the PG layer is known to be fixed, the exact position is not known. In this situation, PG layer prediction can provide a possible location of the PG layer which, when preformed on several periplasm spanning proteins from the species of bacteria, could be used to predict the cell-wide position of the cell wall. In the case where the position is known, the prediction can be biased with this value, meaning that systems will be built with the correct position of the PG layer. The PG layer in some bacteria has low curvature due to the action of OpmA and TolR,^27^ thus implying that around proteins, there will be little deviation from the average position; hence, a strong bias is applied to the prediction when a guess is supplied.

### Peripheral Membrane Proteins

Orientation of peripheral membrane proteins is generally more challenging than that of other types described in previous sections. However, as long as the prediction is reasonably close to the ground truth, simulations can still be conducted. For peripheral membrane proteins, electrostatics play a more significant role, as membrane association is often mediated by Coulombic interactions.^17^ Furthermore, peripheral membrane proteins such as BAR domains can induce membrane curvature, which affects their orientation. MemPrO was run with additional flags, varying charge, a higher number of starting configurations (-ng 72), and more minimization iterations (-ni 300+) on several peripheral membrane proteins, as shown in Figure 6. Due to their high mobility, peripheral membrane proteins may lack a clear orientation, but MemPrO can still be employed to investigate these proteins and, in many cases, provide a good starting orientation for simulations.

**Figure 6:**
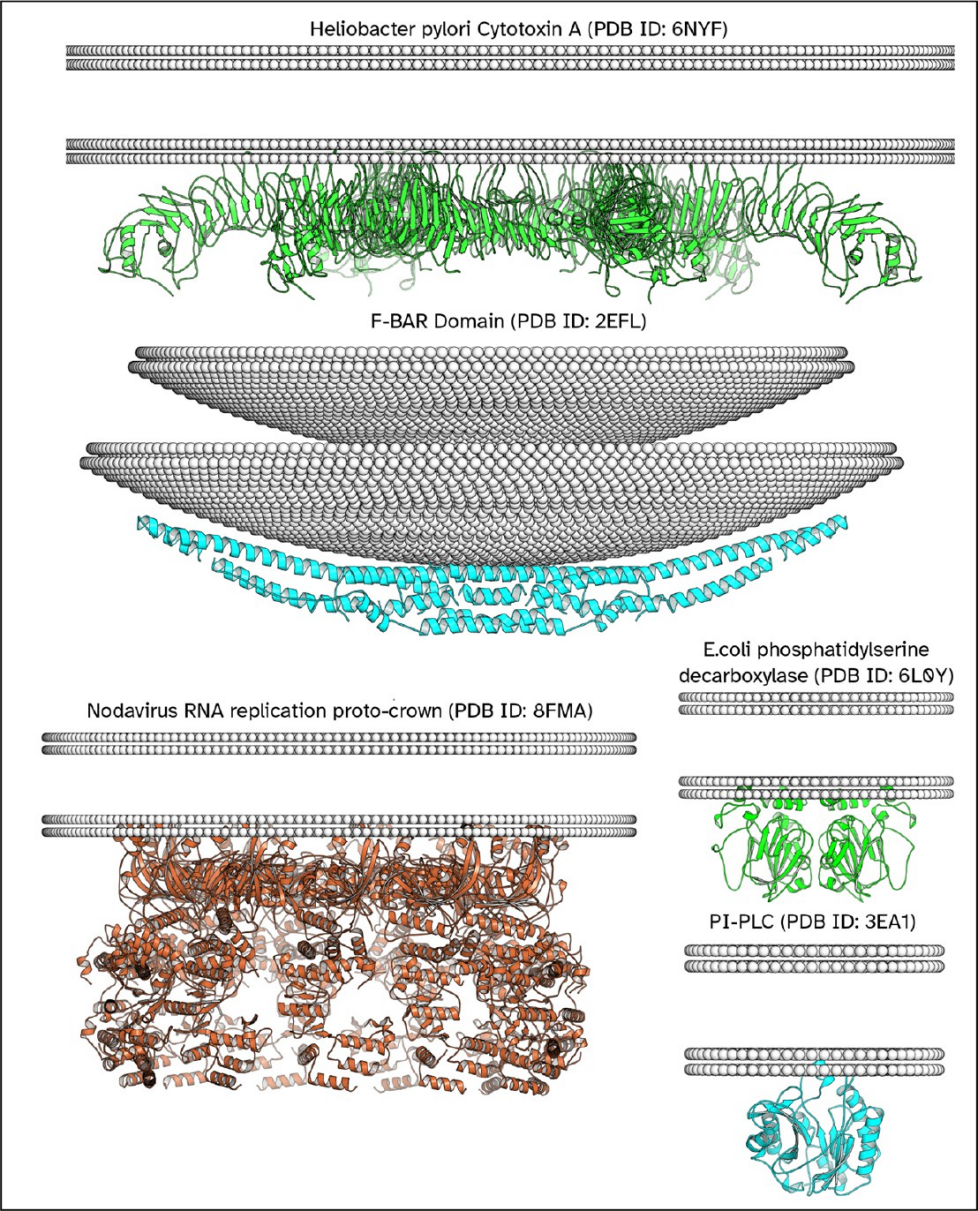
Examples of peripheral membrane protein orientations, with and without use of global curvature prediction. In each of these examples, the orientation agrees with that of the OPM database. In the case of PI–PLC the reference orientation is from the recent paper^28^ (Not shown). The method employed for the positive inside rule results in all positively charged peripheral membrane proteins being placed inside the cell. However, this is simply an artifact of the method.

### Building Simulation Ready Systems

Examples of CG systems are shown in Figure 7 for the different systems that can be oriented with MemPrO. These systems are built using MemPrO directly, meaning all parameters relating to the structure and positions of the protein and lipid bilayer are automatically passed to Insane4MemPrO. The user therefore needs to define only compositional parameters when building the system.

**Figure 7:**
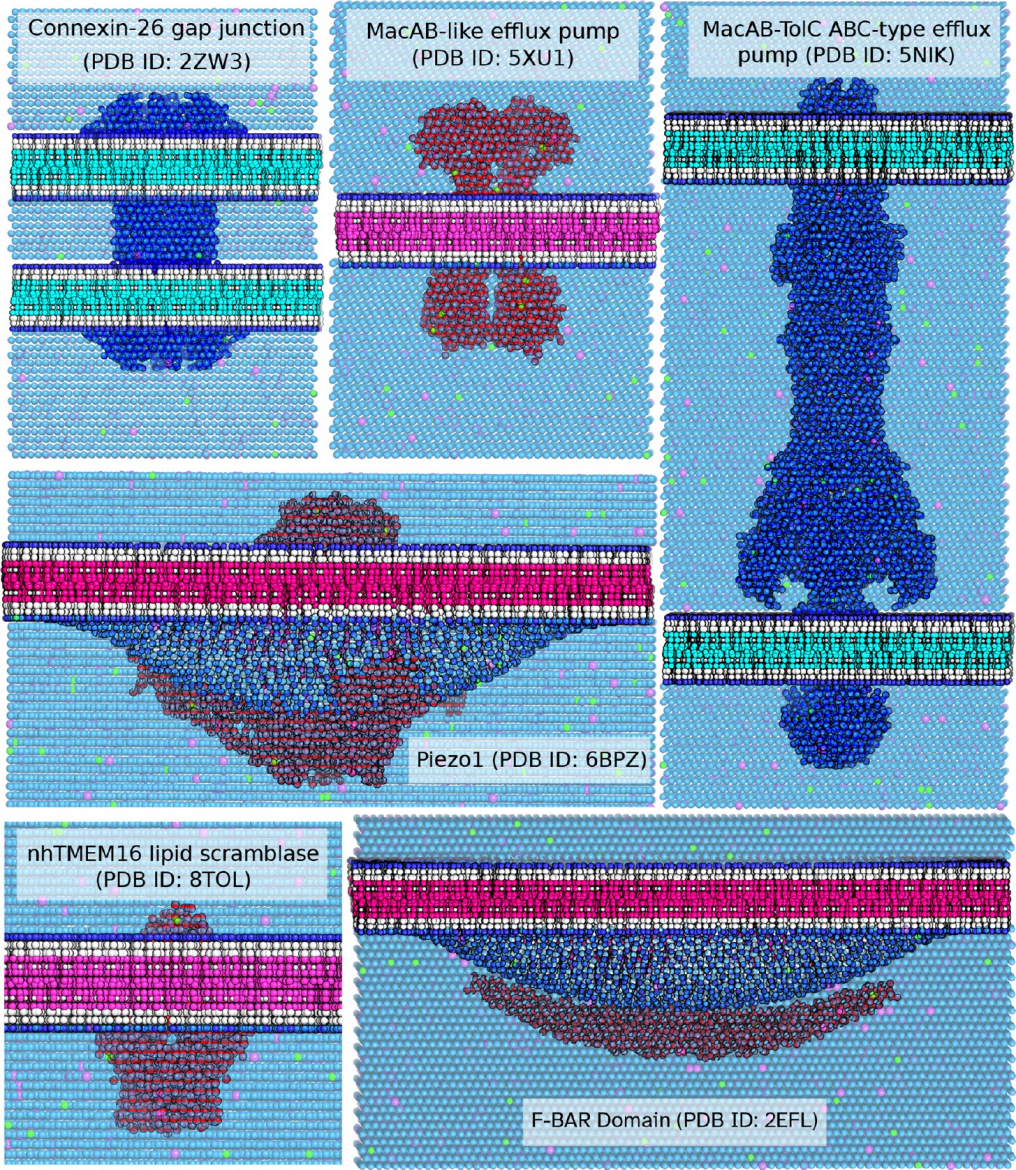
Some example CG systems that were oriented and build using MemPrO. Insane4MemPro can build a large variety of systems. Shown are examples of double membrane systems (2ZW3 and 5NIK), curved membrane systems (6BPZ and 2EFL), and standard single membrane systems (5XU1 and 8TOL).

#### Micelles

When building micelles, Insane4MemPrO will match the shape of the protein, as can be seen in Figure 8a,b. By matching the shape as closely as possible, this ensures that the width of the micelle is constant, which makes it easier to place the correct number of detergent molecules. If instead a circular band was used, this would have an inconsistent width, thus requiring more padding in certain regions, which affects the overall number of detergent molecules. This can be seen most clearly when considering a protein that is far longer in one dimension than the other. During equilibration, detergent molecules will rearrange themselves to be closer to a constant width around the protein, and so starting the simulation as in Figure 8 will lead to less equilibration time.

**Figure 8:**
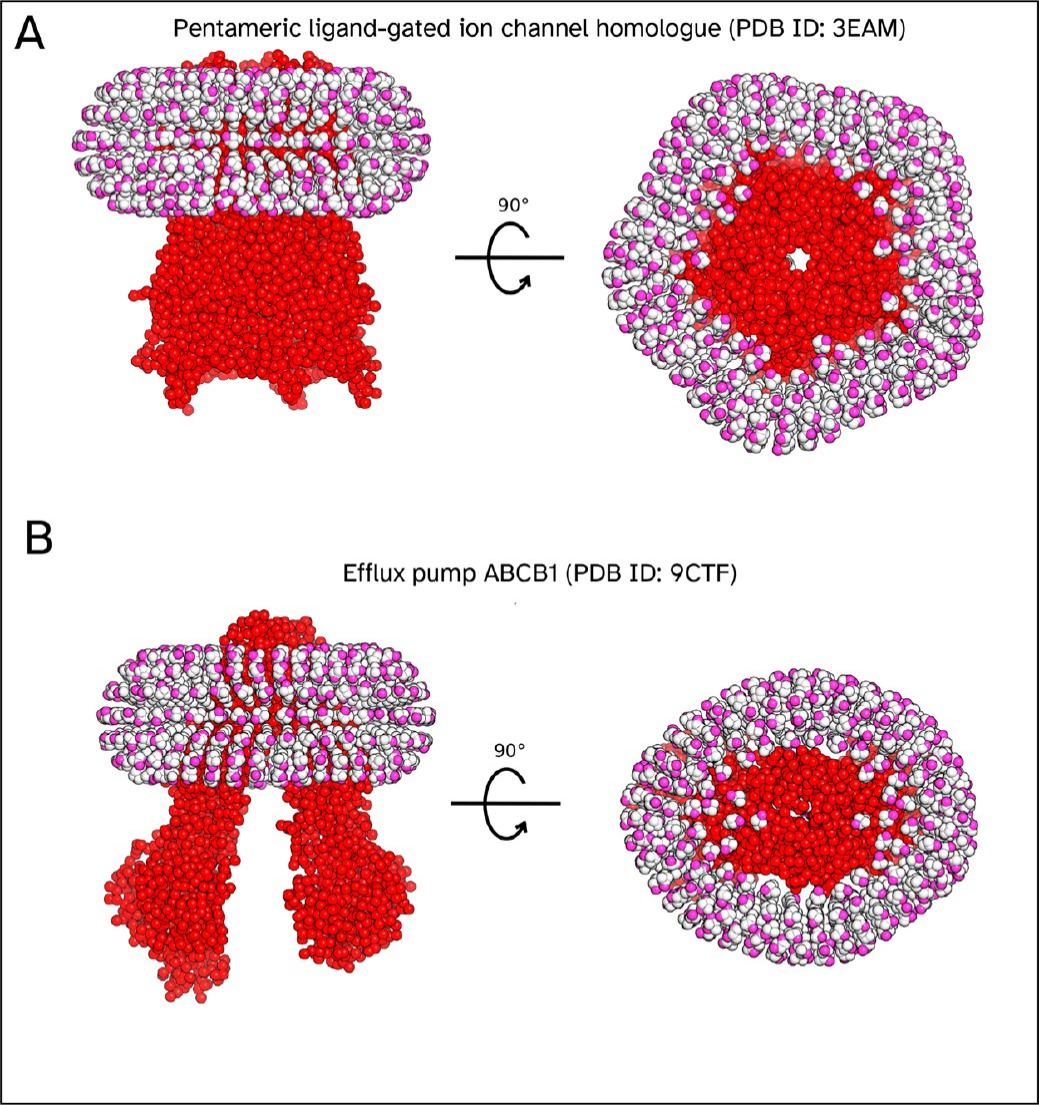
Example of CG systems containing a micelle. (A) Output of Insane4MemPrO was analyzed for 3EAM with a DDM detergent micelle. (B) Output of Insane4MemPrO for 9CTF with a DDM detergent micelle.

#### Peptidoglycan Cell Wall

Figure 9 shows some examples of the PG layer with and without a protein present. Insane4MemPrO can be used directly with MemPrO, in which case the position of the PG layer is automatically passed to Insane4MemPrO. Though the glycan strands within the PG layer are generally depicted as parallel lines, this is almost never the most energetically favorable configuration. Proteins and cross-links will disrupt these lines, leading to a more irregular structure. When building the PG layer, Insane4MemPrO will relax the PG layer using a simple simulation in which the cell size is allowed to change. This ensures that there is zero tension on the PG layer while also letting the glycan strands deform into a more energetically favorable configuration, as can be seen in Figure 9a. The effect of this relaxation step is more evident when there are fewer cross-links or a protein is present, as shown in Figure 9b. This highlights the need for user-defined cross-linking probabilities as the degree of cross-linking heavily affects the structure and mechanical properties^23^ of the cell wall.

**Figure 9:**
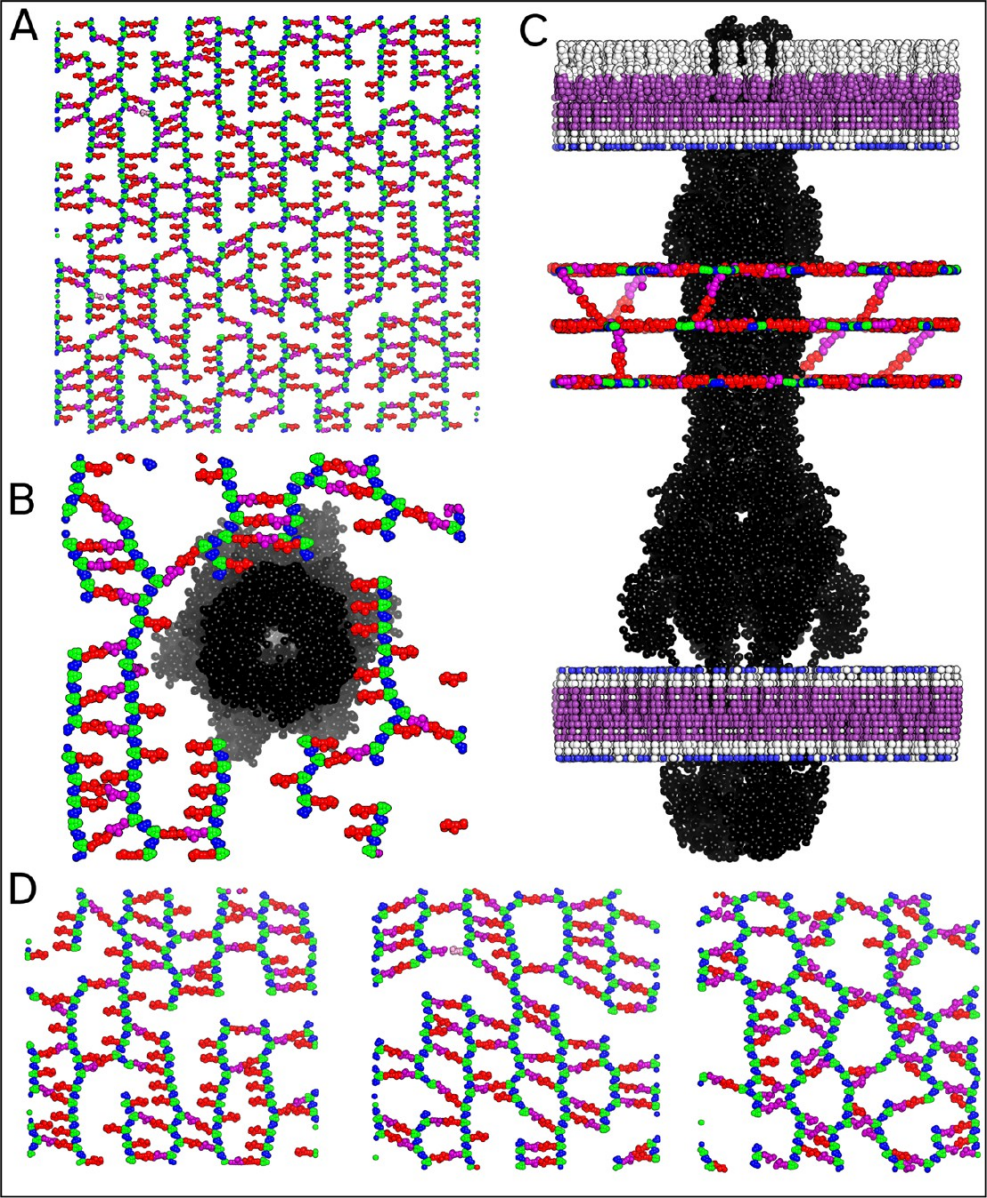
Examples of the PG layer built with Insane4MemPrO. (A) A large peptidoglycan layer. (B) Top view of one PG layer with an efflux pump passing through. (C) Side view of (B) showing all PG layers. (D) Examples of the PG layer are built with different cross-linking settings. Default settings (Left), 80% chance of cross-linking and 80% chance of cross-linking with oligomers enabled with no penalty to oligomer formation (Right).

Simulations of the PG layer in solvent with both an anisotropic and a semiisotropic barostat lead to a constantly shrinking cell. In the paper by Koch et al.,^29^ it was reported that the area of the cell wall could increase by 300% from the relaxed state, implying the possibility that this shrinkage is due to the relaxation of the PG layer. In the case of these simulations, the cross-sectional area of the cell would collapse over a very short time frame, ∼25 ns, until the system became unstable and crashed. As mentioned above, the PG layer was constructed to avoid any overextended bonds being present at the start of the simulation, indicating that any overextension, if at all present, would only be very small. Overall, this implies that the compression is likely not due only to relaxation.

It is hypothesized that the shrinking observed in this case is caused by the nature of the PG layer. When stretched, the PG layer will try to contract; however, when compressed, conformational changes occur, leading to little to no expansion. This presents as a systematic error in the calculation of the pressure, leading to a constant decrease in cross-sectional area due to the action of the barostat. This effect is not limited to computational methods; however, it is possible that the barostat and periodic boundary conditions increase the magnitude of the effect. Similar, but less extreme, examples of this have been reported previously by Beeby et al.,^30^ where the relaxed PG layer would curl, which corresponded with experimental data for Gram-positive bacteria. Given the periodic boundary conditions used in this work, this curling could cause the cell to compress.

#### Validation

Several simulations were run of proteins with PDB IDs: 1A91, 2MJO, 4IKV, 4K3B, 1BT9, 5VPN, 1EK9, 5CFB, and 3RKO. In each case, a simple system consisting of the protein, water, ions, and a POPE lipid bilayer was constructed using the MemPrO workflow. The systems were simulated for 3 × 1 μs and analyzed to verify the stability and accuracy of the resulting systems. In each case, the proteins remained stable within the membrane for the full 1μ*s*, with membrane deformations and lipid/solvent contacts agreeing well with the MemProtMD database entries for the corresponding proteins. This indicates that the systems built with Insane4MemPrO are not biased in some way by the method of construction, hence showing that the method is accurate as well as efficient.

### Analysis of a Cell Cross-Sectional Area With and Without a PG Layer

Figure 10A shows the cross-sectional area of the cell for a simulation containing only a PG layer and solvent/ions. Over a very short time, 25 ns, this area decreases beyond what can be accounted for by relaxation of the system during equilibration. As the PG layer is relaxed during construction using a simple simulation, it was theorized that this shrinking is not just the PG layer relaxing, but also due to an entropic force resulting from how the PG layer responds to extension and compression of the cell.

**Figure 10:**
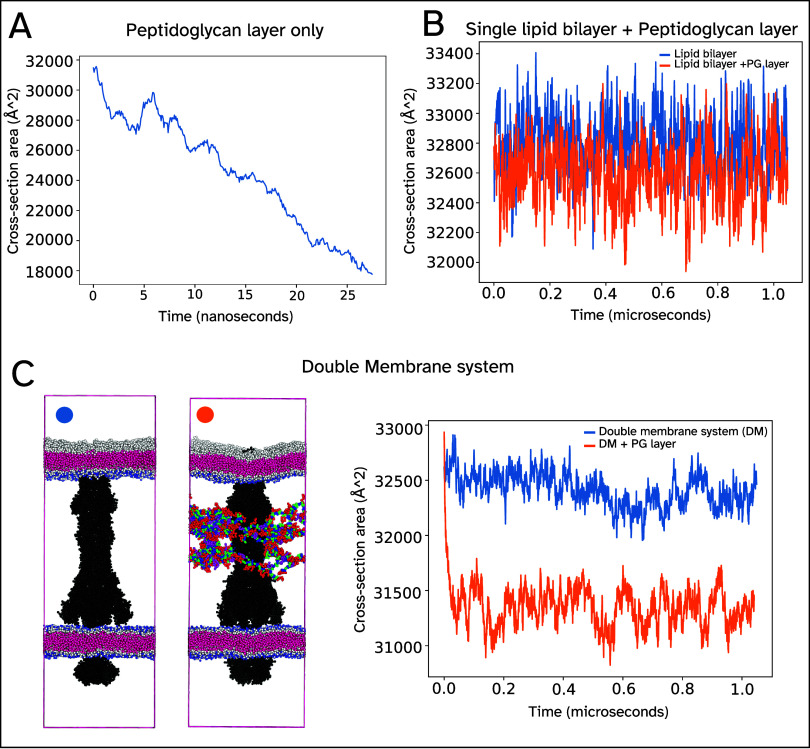
Analysis of the cross-sectional area with and without the PG layer. (A) The PG layer by itself collapses due to entropic forcing. (B) Including a membrane negates the entropic forcing resulting in a stable cell size. This size does not differ significantly to a simulation with the PG layer. (C) As with (B) but for a more realistic system containing a protein (5NIK) and multiple membranes.

If the shrinking is in part due to an entropic force, adding a lipid bilayer would remove the imbalance in response to the cell size changes and thus stop the constant shrinking. In this case, even if some compression of the membrane is still observed, the system should be stable. Figure 10B,C show the cell size during simulation for systems with and without a PG layer. Both systems are stable over 1 μs, and there is only a difference of at most approximately 2% between the cross-sectional areas in both cases, implying that the initial shrinking was indeed due to this entropic forcing. Including a protein causes greater compression, possibly as a result of entropic forcing due to collisions between the protein and the PG layer. The change in the cross-sectional area of the membranes is consistent with the compression observed due to a relaxed PG layer in the paper by Ryoo et al.^31^ This analysis implies that the simulation of the PG layer by itself, therefore, needs either an isotropic barostat or surface tension coupling to provide a restorative force to the compression.

## Summary

Two main methods were presented in this paper. The first of these was MemPrO, a set of tools for the orientation of membrane proteins. MemPrO was shown to be of comparable accuracy to the current state-of-the-art orientation methods. Examples of orientation on a range of different systems, such as double membrane and globally curved systems, were shown, highlighting the versatility of MemPrO.

The second method introduced was Insane4MemPrO, a CG system builder based on *insane* that works in conjunction with MemPrO to build CG systems from orientation prediction, while also functioning as a standalone method for building general CG systems. Some additional features of Insane4MemPrO, such as building micelles around proteins and constructing a zero-tension PG layer that can be used to construct full CG systems of periplasm-spanning proteins in Gram-negative bacteria, were showcased. Further analysis was performed on the nature of the PG layer in CG simulations, showing how entropic forces can affect simulations of the PG layer by itself.

Overall MemPrO and Insane4MemPrO provide an excellent tool-set for the investigation of membrane proteins by simplifying the process of creating CG systems, while also providing a wealth of information on the orientation, facilitating further analysis of more complex proteins.

## Supplementary Material



## Data Availability

MemPrO and Insane4MemPrO are publicly available for use at: https://github.com/pstansfeld/MemPrO. All protein structures used are available, using PDB ID given, on: https://www.rcsb.org/. The mpstruc database used for evaluation can be found on: https://blanco.biomol.uci.edu/mpstruc/.

## Notes

Additional details on the MemPrO/Insane4MemPrO methodology and further investigation of orientations (PDF)
