# Supplementary material for: MemPrO: A Predictive Tool for Membrane Protein Orientation
Source: J Chem Theory Comput. 2025 Dec 23;22(1):638–52. doi: 10.1021/acs.jctc.5c01433 (PMC12805567; doi:10.1021/acs.jctc.5c01433)
Supplement: Supplementary file 1 [file ct5c01433_si_001.pdf]

MemPrO: A Predictive Tool for Membrane Protein Orientation

Matyas Parrag[†] and Phillip J. Stansfeld[*,†,‡]

†*School of Life Sciences, University of Warwick, Coventry, UK, CV4 7AL*

‡*Department of Chemistry, University of Warwick, Coventry, UK, CV4 7AL*

E-mail: phillip.stansfeld@warwick.ac.uk

Phone: +44 (0)24 765 23864

Double membrane prediction

Varying (θ, ϕ) alongside D would prove too expensive, so D needs to be approximated, similar to z. Since these proteins likely contain two hydrophobic regions, neither $Z(\theta, \phi)$ or $Z'(\theta, \phi)$ will provide a suitable approximation. Therefore, a more sophisticated method is required for approximating both z and D. To achieve this, the orientation of the protein needs to be guessed, which can be done by leveraging the geometry of a protein that passes through two membranes. The method is as follows:

Let $\mathbf{p_f}$ denote the position of the CG bead furthest from the center of mass of the protein, and let $d_f = |\mathbf{p_f}|$. Define the set P as:

$$P = \{\mathbf{x} \,|\, \mathbf{x} \in B, |\mathbf{x}| > 0.85 \times d_f\} \tag{1}$$

where B is the set of positions of all CG beads on the protein's surface. Define M as:

$$M = \{\mathbf{x} | \mathbf{x} \in B \ , \ |\mathbf{x} - \mathbf{p_f}| < d_f\} \tag{2}$$

The intersection $P \cap M$ will contain only points at one end of the protein. If the protein can be approximated as a long cylinder of varying radius, then the average direction V of points in $P \cap M$ will point along the Z-axis of the cylinder. For proteins that span two membranes, this assumption is reasonable. In cases where this assumption does not hold, V will still be close to the true direction. The protein, Pr, is rotated such that V aligns with the Z-axis and is split into two sets of beads:

$$Pr_1 = \{z_b > z_{\text{com}} | b \in Pr\} \tag{3}$$

and

$$Pr_2 = \{z_b <= z_{\text{com}} | b \in Pr\} \tag{4}$$

where z_b is the z-position of bead b, and z_{com} is the z-coordinate of the center of mass of Pr.

If V is within tolerance t of the true direction (with t depending on the length of Pr), each of Pr_1 and Pr_2 will contain one of the two hydrophobic regions. Denoted by $z_1^{'}$ and $z_2^{'}$, the approximation of the insertion depth using $Z^{'}(\theta, \phi)$ for Pr_1 and Pr_2 respectively. Then, define:

$$z_p = \frac{z_1^{'} + z_2^{'}}{2} \tag{5}$$

and

$$D_p = |z_1^{'} - z_2^{'}|, \tag{6}$$

where z_p and D_p are the estimations of the insertion depth and the distance between the membranes, respectively.

To construct the set of initial configurations, G, (θ_i, ϕ_i) is generated using a Fibonacci

spiral lattice as before. In this case $\theta = 0$ and $\phi = 0$ corresponds to the rotation when V is aligned with the Z-axis. z and D are not varied across i, so the set of initial configurations is $G = \{(z_p, \theta_i, \phi_i, D_p)\}$. When calculating the potential, the distance between the two membranes D is multiplied by $\cos(\theta)$ to ensure that the hydrophobic regions always intersect the membranes.

Coarse grained molecular dynamics

All simulations were run using GROMACS 2023[1] with a timestep of 7 fs for simulations including the PG layer and 20 fs otherwise. The PG layer and lipid bilayers were build using Insane4MemPrO and solvated with water and 0.15 M NaCl to neutralise the system. An elastic network of 500 kJ mol-1 nm-2 was applied between all backbone beads between 0.5 and 0.9 nm. Electrostatics were described using the reaction field method, with a cut-off of 1.1 nm using the potential shift modifier and the van der Waals interactions were shifted between 0.9-1.1 nm. The systems were first energy minimized by steepest descent algorithm to 1000 kJ mol-1 nm-1, further equilibration was preformed depending on the system before simulation. The temperature and pressure were kept constant throughout the simulation at 310 K and 1 bar respectively, with the PG layer, protein, lipids and water/ions coupled individually to a temperature bath by the V-rescale method[2] and a semi-isotropic C-rescale barostat.[3]

Atomistic molecular dynamics

All-atom simulations of the PG layer were performed without position restraints for a total of 200 ns over 3 repeats. In all cases a 1 fs timestep was used, in an NPT ensemble with V-rescale temperature coupling at 310 K[2] and a semi-isotropic C-rescale barostat at 1 bar, with the PG layer, protein, water/ions and, if included, lipids coupled individually.[3] Electrostatics were described using PME, with a cut-off of 1.2 nm and the van der Waals interactions

were shifted between 1-1.2 nm. The TIP3P water model was used, the water bond angles and distances were constrained by SETTLE.[4] H-bonds were constrained using the LINCS algorithm.[5]

Furthest initial configuration

Each initial configuration is defined by two angles θ and ϕ. These values can be represented as points on the surface of a unit sphere S. Let H be the set of points on S corresponding to the set of initial configurations G. G is unbiased if the set H is evenly distributed on S. It is well known that a Fibonacci spiral lattice can be used to generate a evenly distributed set of points on a sphere. Using this a H can be created which corresponds to a G which is unbiased as desired.

As there is z-axis symmetry in the bilayer only one hemi-sphere, $S^{'}$, is used. The set H is evenly distributed on $S^{'}$ meaning each point can be assumed to have equal area per point hence, the area per point is approximately equal to $\frac{2\pi}{N}$, where N is $|H| = |G|$. The area of a circle on the surface of a sphere with radius r along the surface of $S^{'}$ is given by $2\pi(1 - cos(r))$. This gives the equality

$$1 - \frac{1}{N} = cos(r) \tag{7}$$

, which then results in

$$r = cos^{-1}(1 - \frac{1}{N}) \tag{8}$$

, where r represents the distance between adjacent points in H. This then corresponds to the furthest angle deviation in radians possible between some configuration and an element of G.

Validation

MemPrO takes as an optional argument the number of initial configurations (N_G) and the number of minimisation iterations (N_I).

Increasing N_G and N_I will improve accuracy of predictions at the cost of computational efficiency with diminishing returns at higher values of N_G and N_I.

As the set of initial configurations G is unbiased, there will always be a starting configuration within $cos^{-1}(1-\frac{1}{N_G})$ radians of the true configuration, see SI section 1 for a derivation. This corresponds to $\sim$ 40 degrees for $N_G = 4$ and $\sim$ 13.5 degrees for $N_G = 36$. Even with the minimum value of $N_G = 4$ good results are possible with very few minimisation iterations.

Figure S1A shows the average deviation, in degrees, from MemProtMD[6] across a dataset of 600 proteins randomly selected from the mpstruc database[7] as iterations are increased from 1 to 150. OPM-PPM3 and Memembed are shown for context. Due to the error inherent in the calculation of angle deviation, and the variance in the actual orientation the differences between methods are negligible.

There are diminishing returns after approximately 150 iterations. Note that here all input configurations are at 90 degrees from the correct orientation. This is in order to avoid possible bias from initial orientation while also allowing fair comparison between MemPrO runs with different numbers of iterations. These results justify using $N_I = 150$ as the default number of iterations.

There are two possible sources of randomness in MemPrO. Firstly, the surface finding algorithm has inherent randomness in order to reduce computational cost, and secondly the orientation of the input is used to determine the grid of initial configurations G. To quantify how these two sources of randomness affect the final orientation MemPrO was run 1200 times on 5 randomly selected proteins. For 600 runs the input was randomly rotated, while for the other 600 the input was rotated at 90 degrees from the true orientation.

Figure S1B shows the KDE distributions of the deviation from MemProtMD in degrees. The randomly rotated inputs and 90 degree rotated inputs correspond to the solid and dotted

lines respectively. Little difference can be observed between the KDE graphs indicating that the input does not affect the final orientation. The setup of the initial configurations was designed specifically to avoid bias from the input configuration and the efficacy of this method is highlighted in these results.

In order to provide some comparison, Memembed[8] was also rerun 600 times on each of the 5 proteins. The randomness in Memembed comes from the genetic algorithm used which randomly mutates orientations that preform well. Figure S1C show the KDE distributions as with MemPrO. In this case the distribution is highly dependent on the protein. OPM-PPM3 does not have any randomness in its orientation algorithm, which was verified by re-running it on several proteins.

Orientations with greater than 30 degrees deviation

In the dataset of 1175 alpha helical proteins there were a few cases where one or more of the methods tested deviated by more than 30 degrees. Such deviations can cause a simulation to fail or produce incorrect results. The majority of such fail cases were proteins with less than 3 transmembrane helices, which can be very mobile within the membrane so there may not be a well defined orientation. Some of the proteins in the dataset were miss-folded, had missing atoms or had other defects that have a major impact on the surface hydrophobicity causing the physics based methods to fail. This leaves a small number of proteins in the dataset where an orientation exists but is not found by at least on of the methods.

In the case of MemPrO, 2 distinct proteins failed to orient. The first of these was a gap junction which passes through two membranes. When configured for double membrane orientation this protein was correctly oriented. The second of these was a lipid A transporter with PDB code 6BL6.

Figure S2A shows the orientation found by MemPrO, for 6BL6, compared to the orientation found by OPM-PPM3. Investigating the output of MemPrO shows that the correct

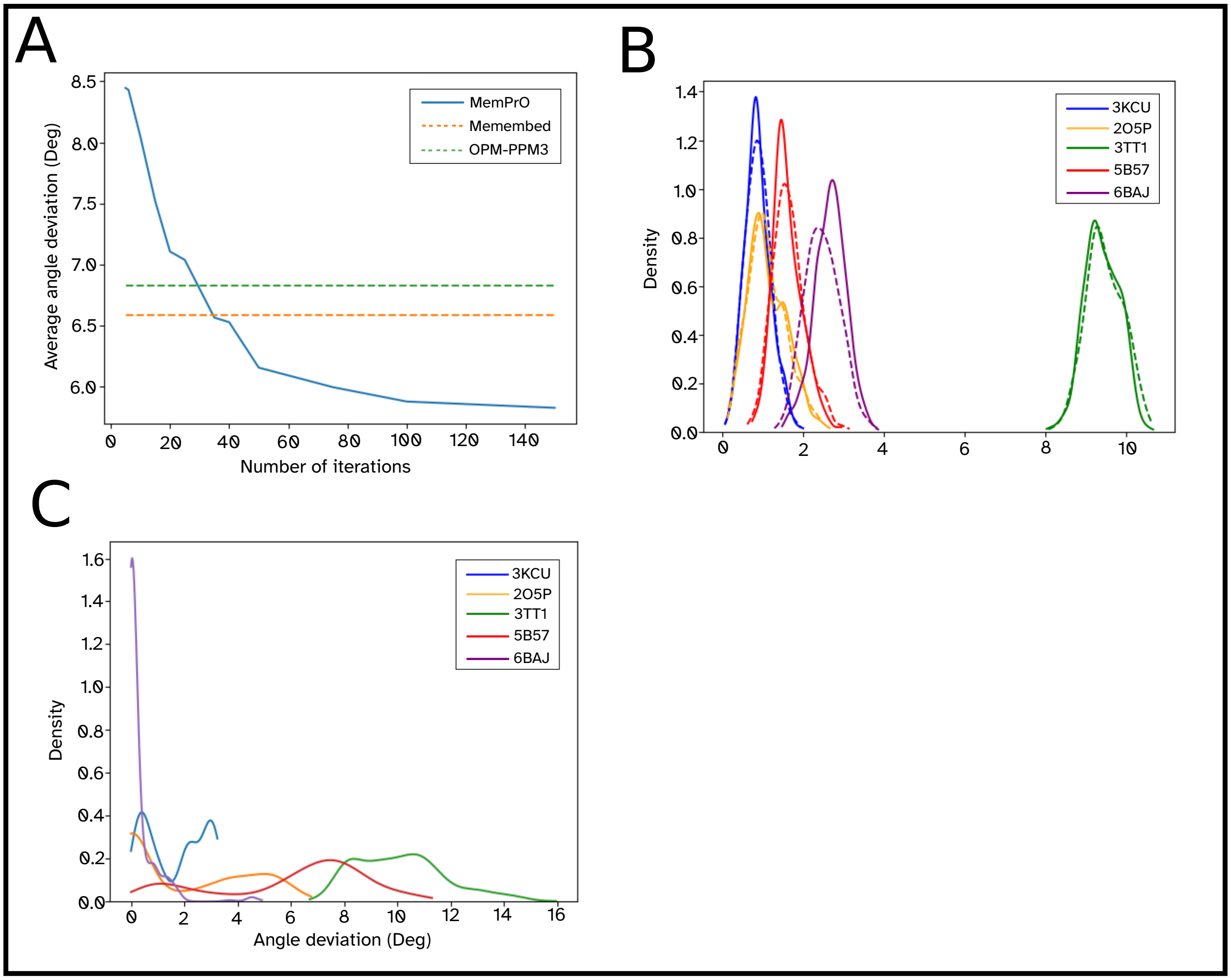

Figure S1: *Graphs showing MemPrO's performance and reliability.* A: Average angle deviation with increasing iterations, Memembed and OPM-PPM3 shown for context. MemPrO's performance shows diminishing returns with more than 150 iterations. B: Distributions of angle deviation over 600 runs with MemPrO, this shows a low variation between orientations indicating a high reliability. C: Distributions of angle deviations over 600 runs for Memembed, the bimodal distributions are a result of the genetic algorithm.

orientation is obtained but ranked below the incorrect orientation. Looking at the Z-position against score graphs of both of these orientations (Figure F) shows that due to the unusual shape of the protein the incorrect orientation lies in a very deep minima. It is difficult to distinguish purely from these graphs, without additional information, which is biologically correct. This example highlights the importance of reporting all minima found.

There were as with MemPrO a few cases where OPM-PPM3 failed to orient. Figures S2C show some of these cases. Investigation of the contribution of each residue to the total score calculated by MemPrO shows the presence of non-hydrophobic residues within the membrane. This would usually indicate missing atoms, however in this case no missing atoms are specified in the PDB file. These non-hydrophobic residues are likely the cause of the failed orientations.

Non-Membrane proteins

MemPrO currently does not distinguish between membrane proteins and non-membrane proteins. MemPrO orients proteins by looking at the environment of each CG-bead on the surface. For an integral membrane protein there is a hydrophobic band which prefers the core of the membrane which drives the orientation. When no such region exists then charge becomes relevant as with some peripheral membrane proteins.[11] When there is no obvious charged region that associates with the membrane, as with soluble proteins, minima still exists but are very shallow and usually correspond to variations caused by individual residues. This can be observed in figure S3. This resembles the way in which a peripheral membrane protein may associate with the membrane.[11] The key differences are in the nature of the minima, in other words the depth and relative score.

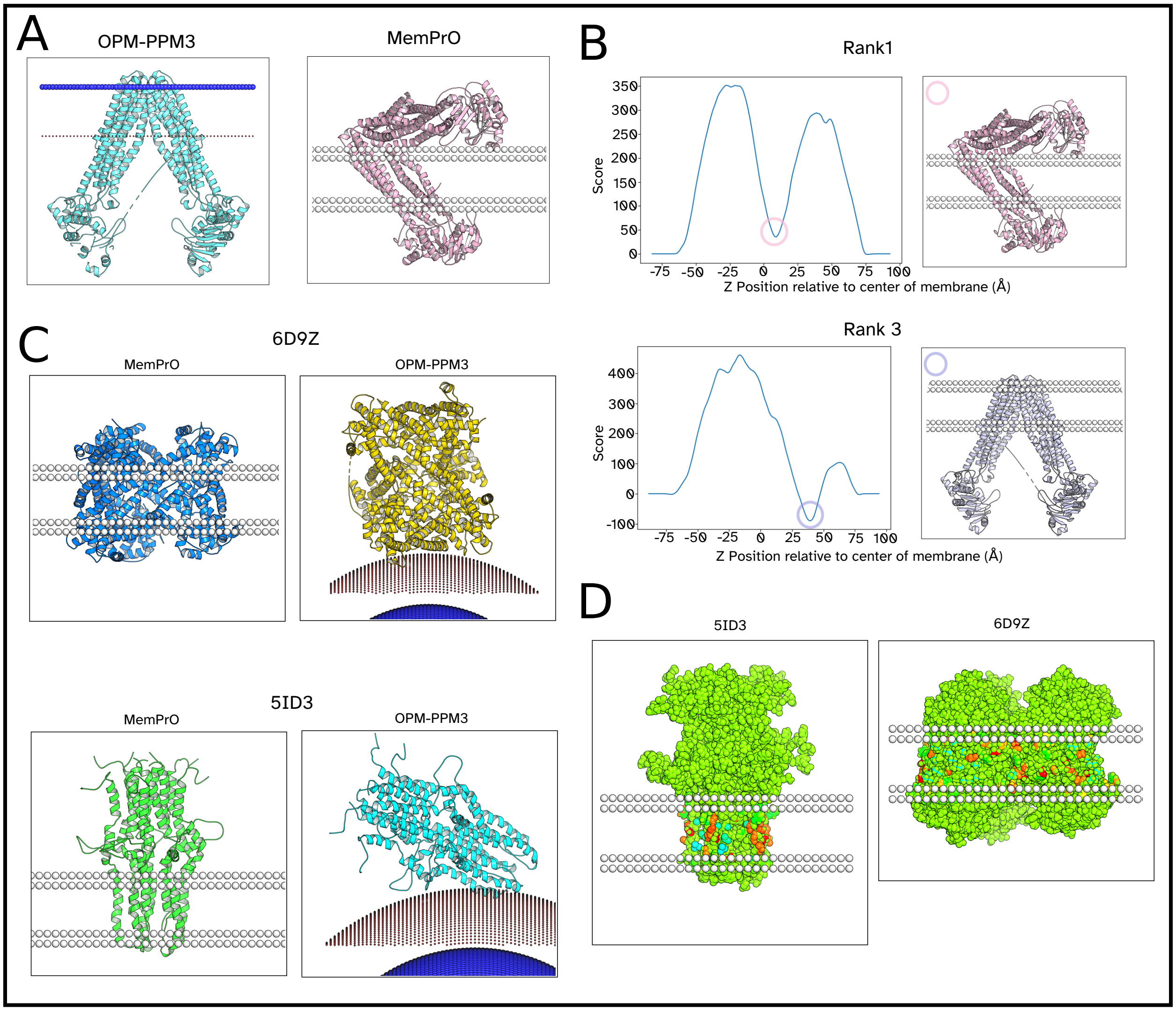

Figure S2: *Examples of proteins where orientation failed.* A: An example of a protein where MemPrO fails. B: Further investigation with MemPrO shows the incorrect orientation is in fact in a deep minima. C: Examples of proteins where OPM-PPM3 fails. D: Contribution of each residue to the total score calculated by MemPrO. This shows the presence of hydrophilic residues within the hydrophobic region, which is causing the issues with orientation.

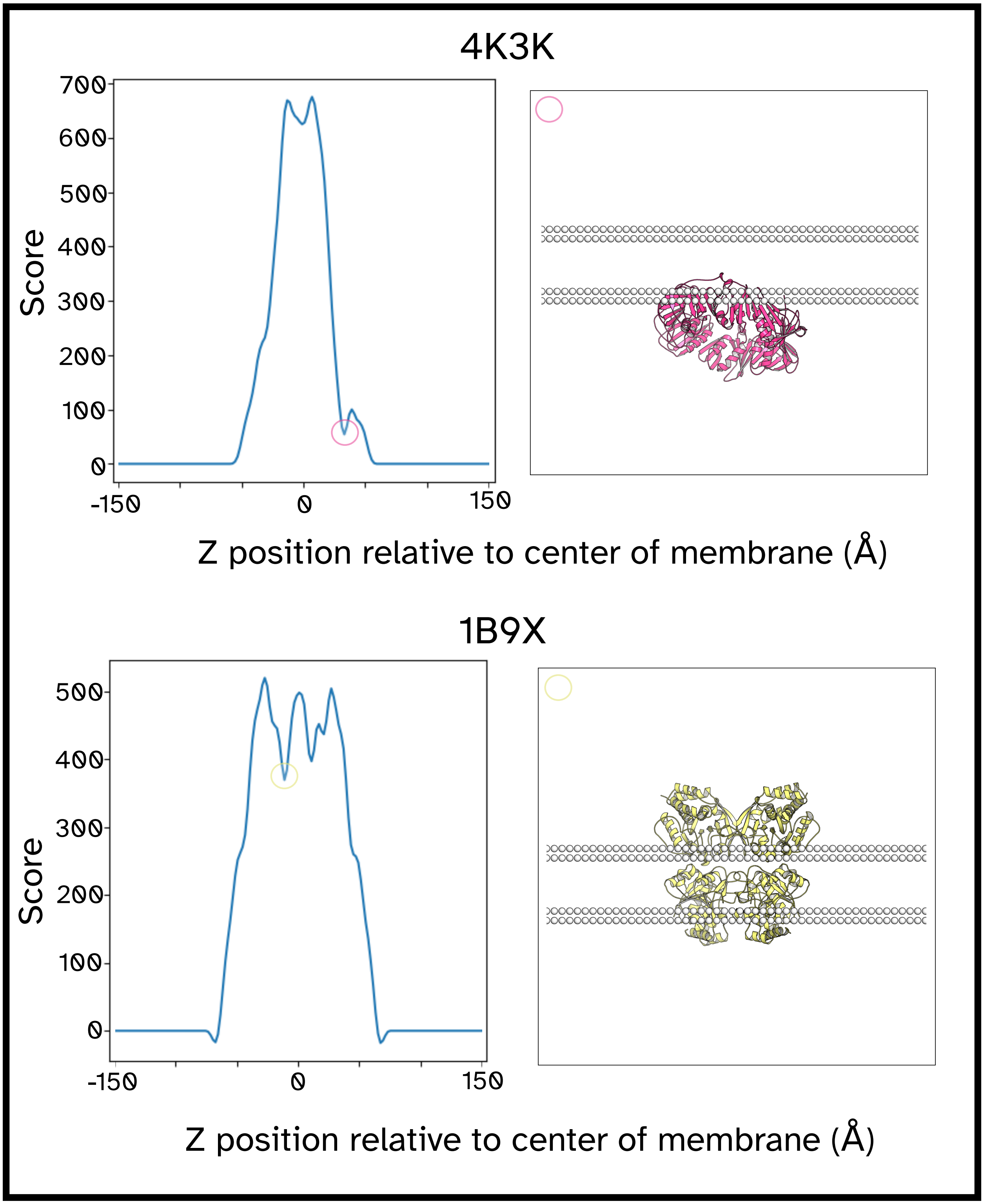

Figure S3: *Examples of soluble proteins.* MemPrO orients soluble proteins, but investigation of score curves shows the minima found are high in score and shallow, indicating low confidence.

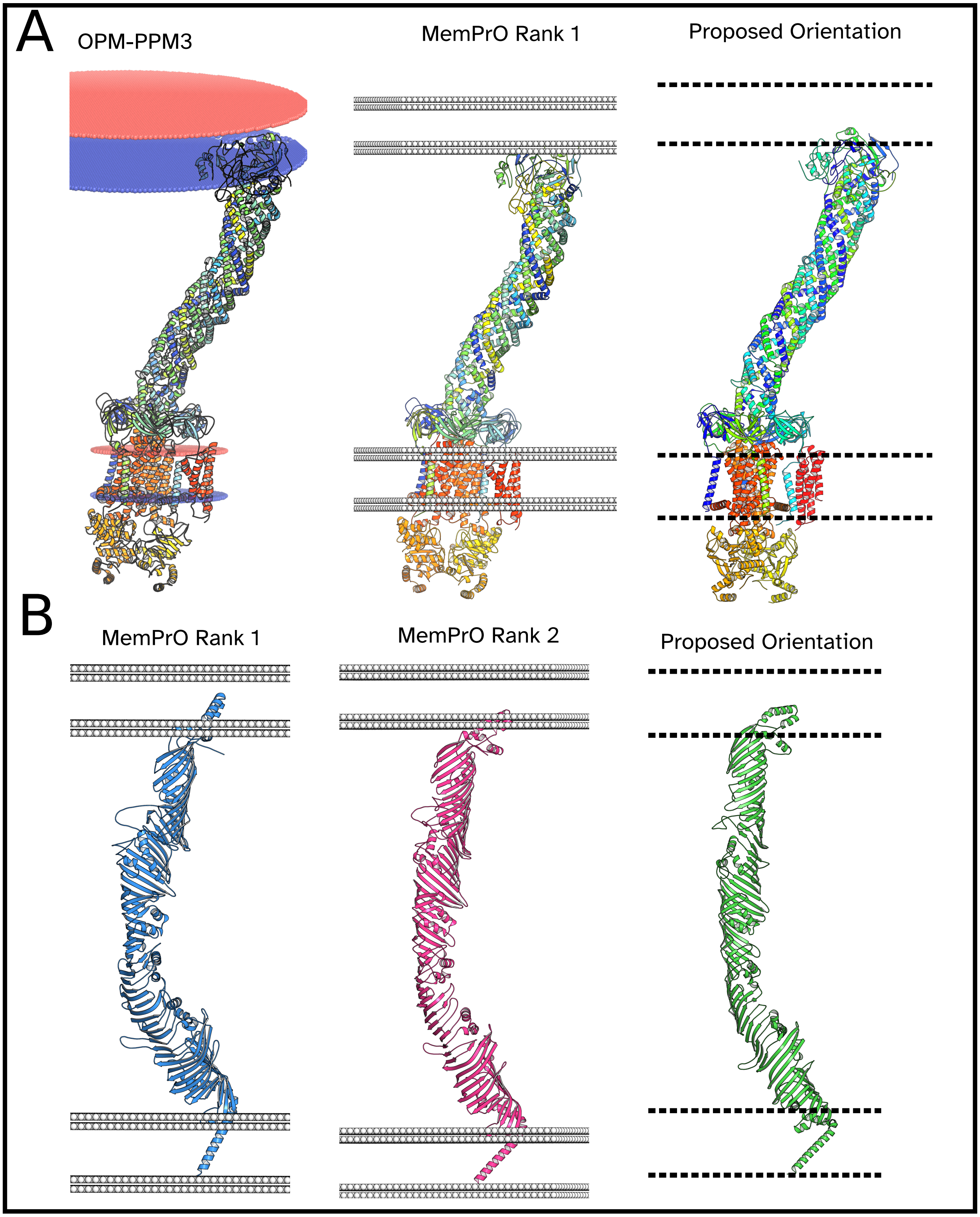

Figure S4: *Further examples of double membrane systems orieted using MemPrO.* A: Orientation of 8FED with MemPrO and OPM-PPM3 compared with orientation proposed in[9] B: YhdP was oriented using MemPrO, the rank 1 and rank 2 were similar in score, with rank 2 corresponding with simulations from.[10]

Other double membrane systems

MemPrO was used to orient a few more examples of double membrane systems, of which one was folded using AlphaFold2.[12] These systems were generally more challenging than those present in the PDB. The first of these was 8FED from the paper.[9] The lowest pseudo potential orientation is shown in figure S4A. This agrees well with the orientation shown in the paper.

Another double membrane system oriented was YhdP from the recent paper.[10] This is a lipid transport with minimal insertion into the membrane. Due to the model consisting of a single chain it was not possible to orient this protein using OPM.

Figure S4B shows that in this case the rank 2 orientation is closer to simulation than rank 1. Rank 1 and Rank 2 are close in potential which indicates more is needed to differentiate between the minima.

Usability

MemPrO is intended to be used as a command line tool, which is to say all of the functions of MemPrO can be preformed by executing a single command line which can be configured through optional flags. Figure S5A shows a schematic of the overall workflow, and Figure S5B shows the general structure of the output of MemPrO for a single membrane orientation.

As MemPrO is purely a terminal based tool all configuration of the output is through optional flags. This is very similar to other command line tools, such as *insane* and Memembed. This approach is slightly less user friendly than creating a graphical user interface (GUI), such as CHARMM-GUI or the web version of OPM-PPM 3.0, however it lends itself better for use in automatic workflows. Additionally MemPrO is intended to be downloaded and used locally, which has benefits and downsides when compared to a web tool. When using a web tool the user has access to the computational power of the servers which host the web tool, which can lead to faster execution times and larger jobs. An issue with relying on

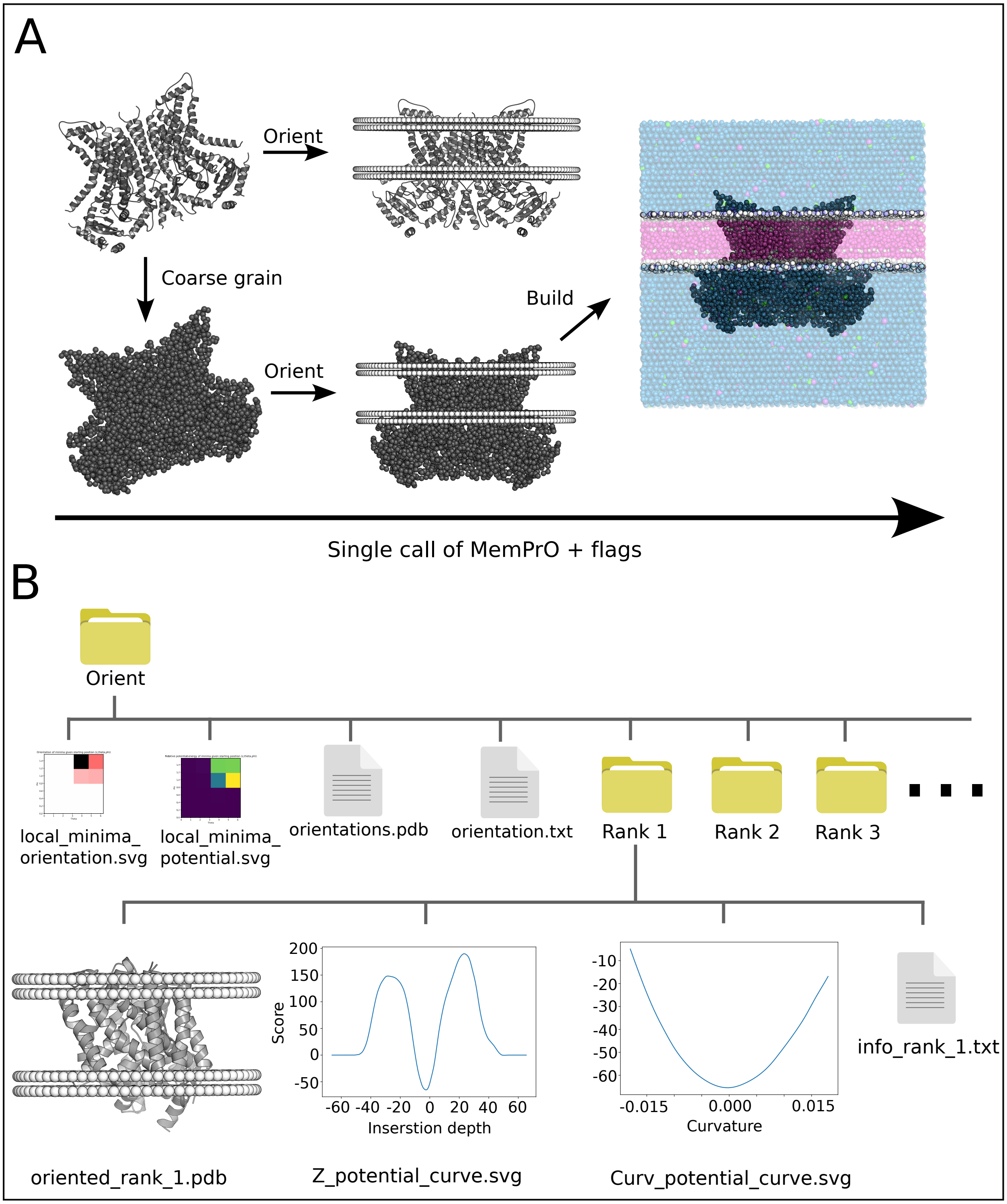

Figure S5: *The MemPrO workflow.* 5a: The general MemPrO workflow consists of orientation followed by building a CG system. All function of MemPrO are run with a single command with flags to configure the outputs. 5b: The general structure of the output of a simple orientation using MemPrO.

external servers is that server load can affect the overall runtime of each task. This includes loading times, and time waiting in queues, which in some cases can become prohibitive even for simple tasks. As compute power is increasing over time and general users have access to more and more compute power, the benefits of using external compute power become less impactful, while the inherent issues that come with it remain the same. MemPrO mitigates this downside of local methods by being relatively lightweight and efficient, hence running in reasonable times on standard desktops/laptops.

MemPrO contains several features which overlaps with many different methods, a summary of all the features included in MemPrO compared to OPM-PPM 3.0, Memembed and CHARMM-GUI are shown in Figure S6.

References

(1) Abraham, M. J.; Murtola, T.; Schulz, R.; Páll, S.; Smith, J. C.; Hess, B.; Lindah, E. GROMACS: High performance molecular simulations through multi-level parallelism from laptops to supercomputers. *SoftwareX* **2015**, *1-2*, 19–25.

(2) Bussi, G.; Donadio, D.; Parrinello, M. Canonical sampling through velocity rescaling. *The Journal of chemical physics* **2007**, *126*.

(3) Bernetti, M.; Bussi, G. Pressure control using stochastic cell rescaling. *Journal of Chemical Physics* **2020**, *153*.

(4) Miyamoto, S.; Kollman, P. A. Settle: An analytical version of the SHAKE and RATTLE algorithm for rigid water models. *Journal of Computational Chemistry* **1992**, *13*, 952–962.

(5) Hess, B.; Bekker, H.; Berendsen, H.; Fraaije, J. E. M. LINCS: A linear constraint solver for molecular simulations. *Journal of Computational Chemistry* **1997**, *18*, 1463–1472.

Features	MemPrO	OPM-PPM 3.0	CHARMM-GUI	MemEmbed
**Orientation**	✓	✓	✓ / X[5]	✓
Single Membrane	✓	✓	✓	✓
Double Membrane	✓	✓[2]	X	X
Global Curvature	✓	✓[3]	X	X
Protein Lipid complex	✓	✓	✓	X
PG layer prediction	✓	X	X	X
Average runtime [8]	145.4 s	718.9 s [4]	– [6]	8.3 s
**Building CG systems**	✓	X	✓	X
Single Membrane	✓	–	✓	–
Double Membrane	✓	–	✓[7]	–
Curved Membrane	✓	–	X	–
Micelles	✓	–	✓	–
PG layer	✓	–	X	–
Nanodisks	✓	–	✓	–
Atomistic	X[1]	–	✓	–
**Overall Workflow**	✓	X	✓	X
Average runtime[9]	184.7 s[10]	–	630 s [11]	–
**CPU scaling**	✓	X[4]	–	X
**Local/Web based**	Local	Both	Web based	Local

1: Only CG systems can be built, however these can easilty be converted to atomistic using CG2AT.

2: Membranes are oriented independantly based on a subset of chains. This can lead to issues with non-parralel membranes and difficulties orienting ceratin periplasm spanning proteins.

3: Global curvature prediction is optional when using the web tool, however when suing the local version it is automatically applied. Always predicting with curvature can lead to overfitting.

4: Values are for the local version of OPM-PPM 2.0/3.0.

5: Orientation is provided by OPM-PPM 2.0, however this is not available for CG systems.

6: Orientation is not standalone and part of an overall workflow. Run times of the orientation are comaparable to OPM-PPM 3.0.

7: Double membrane systems can be contructed from single memabrane components via a multicomponent assembler.

8: Average runtimes are averaged over several proteins with ~10000 atoms, with MemPrO using 9 CPUs.

9: Average runtimes are for the protein with PDB ID 1EK9, with MemPrO using 9 CPUs.

10: This run time includes using Martinize2 to coarse grain a input atomisitc protein for better comparison with CHARMM-GUI. The time is an average over 5 runs.

11: This run time does not include full orientation as OPM-PPM 2.0 was not avaiable for CG systems. The orientation method used instead was to align the principle axis with the Z axis

Figure S6: *A summary of features in MemPrO, OPM, Memembed and CHARMM-GUI martini maker*

(6) Stansfeld, P. J.; Goose, J. E.; Caffrey, M.; Carpenter, E. P.; Parker, J. L.; Newstead, S.; Sansom, M. S. MemProtMD: Automated Insertion of Membrane Protein Structures into Explicit Lipid Membranes. *Structure(London, England:1993)* **2015**, *23*, 1350.

(7) Membrane Proteins of Known Structure. https://blanco.biomol.uci.edu/mpstruc/.

(8) Nugent, T.; Jones, D. T. Membrane protein orientation and refinement using a knowledge-based statistical potential. *BMC Bioinformatics* **2013**, *14*, 1–10.

(9) Chen, J.; Fruhauf, A.; Fan, C.; Ponce, J.; Ueberheide, B.; Bhabha, G.; Ekiert, D. C. Structure of an endogenous mycobacterial MCE lipid transporter. *Nature 2023 620:7973* **2023**, *620*, 445–452.

(10) Cooper, B. F.; Clark, R.; Kudhail, A.; Bhabha, G.; Ekiert, D. C.; Khalid, S.; Isom, G. L. Phospholipid transport to the bacterial outer membrane through an envelope-spanning bridge. *bioRxiv* **2023**, 2023.10.05.561070.

(11) Yeagle, P. L. Membrane Proteins. *The Membranes of Cells* **2016**, 219–268.

(12) Jumper, J. et al. Highly accurate protein structure prediction with AlphaFold. *Nature 2021 596:7873* **2021**, *596*, 583–589.
